# Structural and mutational analyses of the *Leptospira interrogans* virulence-related heme oxygenase provide insights into its catalytic mechanism

**DOI:** 10.1371/journal.pone.0182535

**Published:** 2017-08-03

**Authors:** Anabel Soldano, Sebastián Klinke, Lisandro H. Otero, Mario Rivera, Daniela L. Catalano-Dupuy, Eduardo A. Ceccarelli

**Affiliations:** 1 Instituto de Biología Molecular y Celular de Rosario (IBR), CONICET, Facultad de Ciencias Bioquímicas y Farmacéuticas, Universidad Nacional de Rosario, Rosario, Argentina; 2 Fundación Instituto Leloir, IIBBA-CONICET, and Plataforma Argentina de Biología Estructural y Metabolómica PLABEM, Buenos Aires, Argentina; 3 Department of Chemistry and Ralph N. Adams Institute for Bioanalytical Chemistry, University of Kansas, Lawrence, Kansas, United States of America; Russian Academy of Medical Sciences, RUSSIAN FEDERATION

## Abstract

Heme oxygenase from *Leptospira interrogans* is an important virulence factor. During catalysis, redox equivalents are provided to this enzyme by the plastidic-type ferredoxin-NADP^+^ reductase also found in *L*. *interrogans*. This process may have evolved to aid this bacterial pathogen to obtain heme-iron from their host and enable successful colonization. Herein we report the crystal structure of the heme oxygenase-heme complex at 1.73 Å resolution. The structure reveals several distinctive features related to its function. A hydrogen bonded network of structural water molecules that extends from the catalytic site to the protein surface was cleared observed. A depression on the surface appears to be the H^+^ network entrance from the aqueous environment to the catalytic site for O_2_ activation, a key step in the heme oxygenase reaction. We have performed a mutational analysis of the F157, located at the above-mentioned depression. The mutant enzymes were unable to carry out the complete degradation of heme to biliverdin since the reaction was arrested at the verdoheme stage. We also observed that the stability of the oxyferrous complex, the efficiency of heme hydroxylation and the subsequent conversion to verdoheme was adversely affected. These findings underscore a long-range communication between the outer fringes of the hydrogen-bonded network of structural waters and the heme active site during catalysis. Finally, by analyzing the crystal structures of ferredoxin-NADP^+^ reductase and heme oxygenase, we propose a model for the productive association of these proteins.

## Introduction

Iron is an essential element for all forms of life. Poor bioavailability of free iron is a primary obstacle for successful bacterial colonization of mammalian hosts. To overcome this limitation, pathogenic bacteria have developed several strategies to acquire and utilize host heme-iron [1], including the use of heme-degrading enzymes known as heme oxygenases (HOs). HOs catalyze the oxidative cleavage of heme to biliverdin, carbon monoxide, and ferrous iron, in a reaction that proceeds *via* a complex multistep mechanism [2,3].

The catalytic process of heme degradation starts with the reduction of ferric heme-iron to its ferrous state, followed by the rapid binding of dioxygen to give an oxyferrous complex (Fe^II^-O_2_), which accepts a second electron and a proton, and is transformed to a ferric hydroperoxide (Fe^III^-OOH) intermediate, which reacts with the heme porphyrin ring to give α-*meso*-hydroxyheme. Conversion of α-*meso*-hydroxyheme to verdoheme entails the O_2_- and electron-dependent elimination of the hydroxylated α-*meso*-carbon as carbon monoxide. Verdoheme is then oxidized to biliverdin and free iron (Fe^II^) in a reaction that requires O_2_ and reducing equivalents. Opening of the verdoheme macrocycle occurs *via* a redox reaction with O_2_, which likely binds the verdoheme iron [4] and produces the Fe^III^-biliverdin complex. Ferric biliverdin is further reduced, and ferrous iron is released with the concomitant liberation of biliverdin (see S1 Fig).

It is well known that canonical HOs have an α-helical fold [5], where heme binds in a hydrophobic pocket between the proximal and distal helices. In the ferric, resting state complex, heme is coordinated by a proximal, non-ionized histidine and by a distal water molecule [6]. A distinctive characteristic of HOs is a network of structural waters that extends from the distal ligand to near the enzyme surface, which is thought to function in the delivery of a H^+^ to the nascent Fe^III^-OO^-^ complex to generate the ferric hydroperoxide (Fe^III^-OOH) oxidizing species [6]. Moreover, the network of structural water molecules appears to be involved in conformational dynamics associated with catalysis, which adapts the active site to the changes that take place during the catalytic cycle and enable the rapid propagation of these changes to remote regions of the enzyme [6], with minimum perturbation of secondary structure. These characteristics allow for the stabilization of the distal ligand, which alters its nature several times during the catalytic cycle of heme degradation. Another unique and important feature that contributes to the stabilization of the heme distal ligand is the presence of a highly conserved glycine residue located in the distal pocket, immediately above the heme [5]. This particular region is thought to allow the kinking and flexibility required of the distal helix to accommodate and stabilize the substrate upon binding, and to facilitate the release of the product [7]. Several reports showed that disruption of the hydrogen bonding network of structural waters [6,8–12] or mutation of the conserved glycine residues [7,13,14] result in significant loss of activity, underscoring the importance of this structural region to HO function.

*Leptospira interrogans* is a pathogenic spirochete that causes leptospirosis, a widespread zoonotic disease transmitted by rodents and other animals. Leptospirosis is an important zoonotic cause of morbidity and mortality and has a substantial impact in the poorest regions of the world and in many undeveloped countries. *Leptospira* causes 1.03 million human infections and *ca*. 60,000 deaths each year [15].

The HO enzyme present in *L*. *interrogans* (LepHO) is required for iron utilization when hemoglobin is the sole iron source [16] and is an important virulence factor in the hamster model of infection [17]. Our recent phylogenetic analysis, which showed a divergence between HOs from saprophytic and pathogenic *Leptospira* species [18], supports the possible role of HO in *L*. *interrogans* pathogenesis. We also showed that the redox partner of LepHO is a ferredoxin-NADP^+^ reductase (LepFNR), a plastidic-type enzyme found in this bacterium [18]. The reductase can efficiently deliver the reducing equivalents needed to support the oxidative degradation of heme to biliverdin and free iron without the requirement of a ferredoxin or a secondary reductant. Given the important role played by LepHO in *L*. *interrogans* pathogenesis, there is considerable interest in elucidating its structure and understanding which features of the enzyme may be important to support its function. Moreover, the crucial role played by HOs in some pathogenic organisms renders these proteins as interesting targets for novel antimicrobial agents. In fact, the potential of this strategy has been highlighted by a series of inhibitors of the iron-regulated heme oxygenase of *Pseudomonas aeruginosa* which have activity against clinical isolates of this opportunistic pathogen [19–21].

Herein we report the crystal structure of LepHO-heme at 1.73 Å resolution, which displays distinct features compared to the structures of other structurally characterized HOs. We also carried out a mutational analysis of residue F157, located on the distal site of the heme binding pocket. The catalytic properties of the variants establish that F157 is critical to maintaining an optimum chemical and dynamical environment for the HO reaction, being responsible for a long-range communication from the HO outer fringes to the active site. Finally, by analyzing the crystal structures of LepFNR and LepHO, we propose a model for the association of these proteins during catalysis.

## Materials and methods

### Site-directed mutagenesis and protein preparation

All LepHOs described in this work were purified and reconstituted with hemin according to previously published procedures [18]. The recombinant LepHO enzymes have 7 residues (GHMASGS) in the N-terminus that are left after removing the histidine tag used for purification using metal affinity chromatography. The mutant recombinant genes were generated using the QuikChange II Site-Directed Mutagenesis Kit (Agilent Technologies, Inc) and the pET-TEV vector harboring the LepHO or LepFNR genes. The oligonucleotides designed to introduce the mutations are depicted in S1 Table. All mutations were confirmed by DNA sequencing.

### Spectral characterization of the heme degradation reaction

UV-visible absorption spectra were recorded over the 300–800 nm range using a Shimadzu UV-2450 spectrophotometer at 25°C and quartz cuvettes of 1 cm path length. The heme degradation activity of wild type LepHO and F157 mutants was analyzed using NADPH and LepFNR as electron sources, as previously reported [18]. Assays typically contained 6 μM LepHO-heme, 0.1 mg/mL catalase (Sigma), 300 μM NADPH and 1 μM LepFNR in 25 mM HEPES (pH 7.5). Reactions were started by addition of the reductase and spectral changes were monitored over 30 min. Kinetic analysis of the electron transfer was performed as reported before [18], maintaining the concentration of LepFNR at 0.5 μM and varying the amount of LepHO-heme from 0.5 to 11 μM. When the effect of ascorbate on the single turnover reaction was examined, 5 mM ascorbic acid was added to 6 μM LepHO-heme and the spectra were recorded every 5 min over 1 h. The first reductase-to-heme electron transfer step was analyzed by monitoring the formation of the LepHO-ferrous heme complex using the NADPH/LepFNR system as equivalent donor in an anaerobic glovebox (Coy, MI, USA). All solutions and materials employed in O_2_-free assays were left inside the glovebox overnight, and UV-visible absorption spectra were recorded using a UV-vis Ocean Optics spectrophotometer installed in the anaerobic chamber. The anaerobic ferric heme complex of wild type or F157I LepHO was reduced by addition of reduced LepFNR (5:1 LepFNR:LepHO molar ratio), which had been previously prepared with an equivalent of NADPH, and spectra were recorded until no further changes were observed. The resultant anaerobic solution of the ferrous heme complexes was removed from the glovebox and bubbled with air to form the corresponding oxyferrous complexes. The autoxidation rates of the oxyferrous complexes were determined by recording the absorption spectra at 30 s intervals during 10 min.

### HPLC and LC-MS analysis

The extraction of LepHO reaction products obtained while employing the NADPH/LepFNR system was performed as described previously [18]. To analyze if verdoheme was accumulated during heme turnover, 5% (v/v) pyridine was added to the reaction mixture and the products were extracted with chloroform. After evaporation of the solvents, the solid residue was dissolved in 5% (v/v) pyridine, and the spectrum was recorded. Also, dry samples were resuspended in methanol/water (85:15, v/v) and analyzed using an Agilent 1200 HPLC system, coupled to a G1314C VWD UV detector and a micrOTOF mass spectrometer (Bruker Daltonics, Germany) operating in the positive-ion mode.

### Aerobic and anaerobic reactions of LepHO-heme complex with H_2_O_2_

Reagent grade 30% H_2_O_2_ was used to prepare fresh 1 mM working solutions, whose concentrations were quantitated by measuring their absorbance at 240 nm (ε_240_ = 43.6 M^-1^ cm^-1^) [22]. A solution of the ferric heme complex of wild type LepHO or F157 mutant (6 μM) was incubated with one equivalent of H_2_O_2_ in the presence of O_2_, and the reaction was monitored with UV-visible spectrophotometry until no further changes were observed. To analyze the coupled oxidation under O_2_-free conditions, the same reaction was carried out in the glovebox.

### Crystallization of the LepHO ferric heme complex

Crystallization conditions for mutant LepHO-heme were screened using the Crystal Screen kit from Hampton Research (Aliso Viejo, CA) in the hanging-drop vapor diffusion method configuration. The protein concentration was 10 mg/mL in 20 mM potassium phosphate buffer pH 7.4. In a typical experiment, 1 μL of the protein was mixed with the same volume of the reservoir solution and equilibrated against 0.5 mL of the reservoir solution. Crystals were grown in 8 conditions after one day at 293 K. Exploration of similar conditions established the best crystals as thin dark brown/reddish prisms grown when 0.1 M sodium citrate pH 6.0 containing 0.2 M ammonium acetate and 24% (w/v) PEG 4000 was used as crystallization solution. Crystal samples of the LepHO ferric heme complex were cryoprotected in an artificial mother liquor, to which PEG 400 had been added to a final concentration of 10% (w/v) to protect the sample from crystalline ice formation on flash cooling, and then cryocooled in liquid nitrogen using Hampton Research loops.

Native X-ray diffraction data from a single crystal were collected at 100 K on a Bruker D8 QUEST microfocus diffractometer equipped with a PHOTON 100 CMOS detector for approximately one day. The complete dataset was then processed to a maximum resolution of 1.73 Å in the orthorhombic *P*2_1_2_1_2_1_ space group with XDS [23]. A total of 5% of the reflections were separated at that stage for cross-validation purposes. Detailed information on data collection parameters and processing statistics are shown in S2 Table.

The LepHO-heme structure was solved by the molecular replacement method using the MrBUMP program [24] from the CCP4 package [25]. For this purpose, the HO-1 from *Synechocystis* sp. (PDB ID: **1WE1**) [26] was selected as search model, and the procedure continued by automated modification of the probe and molecular replacement search both with MolRep [27]. A single polypeptide chain was found in the asymmetric unit with proper crystal packing. Next, several cycles of manual model building and restrained refinement were performed with Coot [28] and Buster [29], respectively. Detailed information of the refinement statistics is presented in S2 Table. The final model was validated with MolProbity [30] and then deposited at the Protein Data Bank under the code 5KZL together with its structure factor amplitudes. Structures were represented using PyMOL Molecular Graphics System V 1.8, Version 1.8 Schrödinger, LLC, USA. Electron density maps were plotted with Phenix [31].

## Results

### Generation of a LepHO mutant to increase the likelihood of crystallization

As early attempts to crystallize the wild type LepHO ferric heme complex were unsuccessful, ternary structure homology models were elaborated with I-TASSER [32] and the available coordinates of HO-1 (PDB ID: **1WE1**) and 2 (PDB ID: **WOW1**) from the cyanobacterium *Synechocystis* sp. PCC6803 [26,33]. These structures were chosen as seeds because of their high sequence identity and similarity to LepHO (0.48), allowing coverage of 99% of the models. In both simulations, the C-terminal end of LepHO protrudes from the globular part of the molecule (S2 Fig, depicted in a dark green and orange), suggesting that this region contains an extended, and probably dynamically disordered segment. Since the presence of disordered structural regions can stymie the growth of protein crystals, we truncated the C-terminal end. To this end, the LepHO-C26S-stop variant was generated by introducing two mutations; the codon for residue E206 was replaced with a stop signal, and the codon for the C26 was replaced by one coding for serine.

C26 is the only cysteine in the LepHO protein sequence and modeling indicates that is on the protein surface. Consequently, the variant, which lacks the last 20 C-terminal residues, ends in L205; C26 was replaced for alanine to prevent inter-molecule disulfide formation thus facilitating isolation and purification (S2 and S3 Figs). LepHO-C26S-stop shows the expected electrophoretic mobility corresponding to a ~24 kDa protein in SDS-PAGE gels (S4A Fig) and electrophoresis in non-denaturing PAGE gels showed that the wild type and mutant enzymes are monomeric in solution (S4B Fig). Reconstitution with heme showed that the mutant incorporates the prosthetic group efficiently, with the corresponding holo-enzyme displaying a UV-Vis spectrum identical to that of the wild type LepHO, with peaks at 403, 500 and 630 nm.

The enzymatic activity of the wild type and mutant LepHO enzymes was compared by following the rate of heme degradation with the aid of electronic absorption spectroscopy using NADPH and LepFNR as electron donors. The reaction proceeds through several intermediate species that can be observed spectrophotometrically because of their unique spectral signatures (S1B Fig). The results show that incubation of the mutant with LepFNR and NADPH causes a decrease in the intensity of the absorption at 403 nm, accompanied by a shift to the Soret band to 410 nm and the emergence of two peaks at 538 and 575 nm, which are identical to those observed with the wild type enzyme. These spectral changes are consistent with the formation of the oxyferrous intermediate, which is then converted to biliverdin, as is evident in the absorbance decay at 538 and 575 nm and the emergence of an absorption band at 680 nm for the mutant and the wild type proteins (S4C and S4D Fig). We have previously determined by ^1^H NMR spectroscopy and HPLC analysis that the product of LepHO heme degradation is the α-isomer of biliverdin [18]. From these data, it can be concluded that neither the replacement of C26 by serine nor the removal of the 20 residues from the C-terminal, significantly alter the capability of the enzyme to degrade heme.

### High-resolution structure determination of the LepHO-C26S-stop ferric heme complex

The LepHO-heme crystal structure was solved by the molecular replacement method using HO-1 from *Synechocystis sp*. (PDB ID: **1WE1**) as search model. The structure was refined to an R factor of 0.185 and a free R factor of 0.224 at 1.73 Å maximum resolution (S2 Table). Ramachandran statistics show that 99% of the residues lie in the favored region of the plot, without outliers. There is one molecule of LepHO in the asymmetric unit and the final model bears 1687 non-hydrogen protein atoms, 43 atoms corresponding to heme and 219 solvent atoms. Although mass spectrometry corroborated the presence of an intact protein (not shown), there was no observable electron density for the first seven N-terminal residues in the model (GHMASGS), probably due to conformational disorder in this region; these residues, which do not correspond to the native sequence, were left after removing the histidine tag included for affinity chromatography purification. The rest of the polypeptide chain presents continuous density that is consistent with the good resolution of the diffraction data. A total of four side chains were observed in alternate conformations and modeled accordingly, following the 2mFo-DFc electron density map. Diffraction parameters and statistics are summarized in S2 Table. To facilitate comparison with other HOs, the amino acid residues in the recombinant LepHO are numbered according to the sequence of the wild type enzyme, starting with methionine at position one.

**Fig 1:**
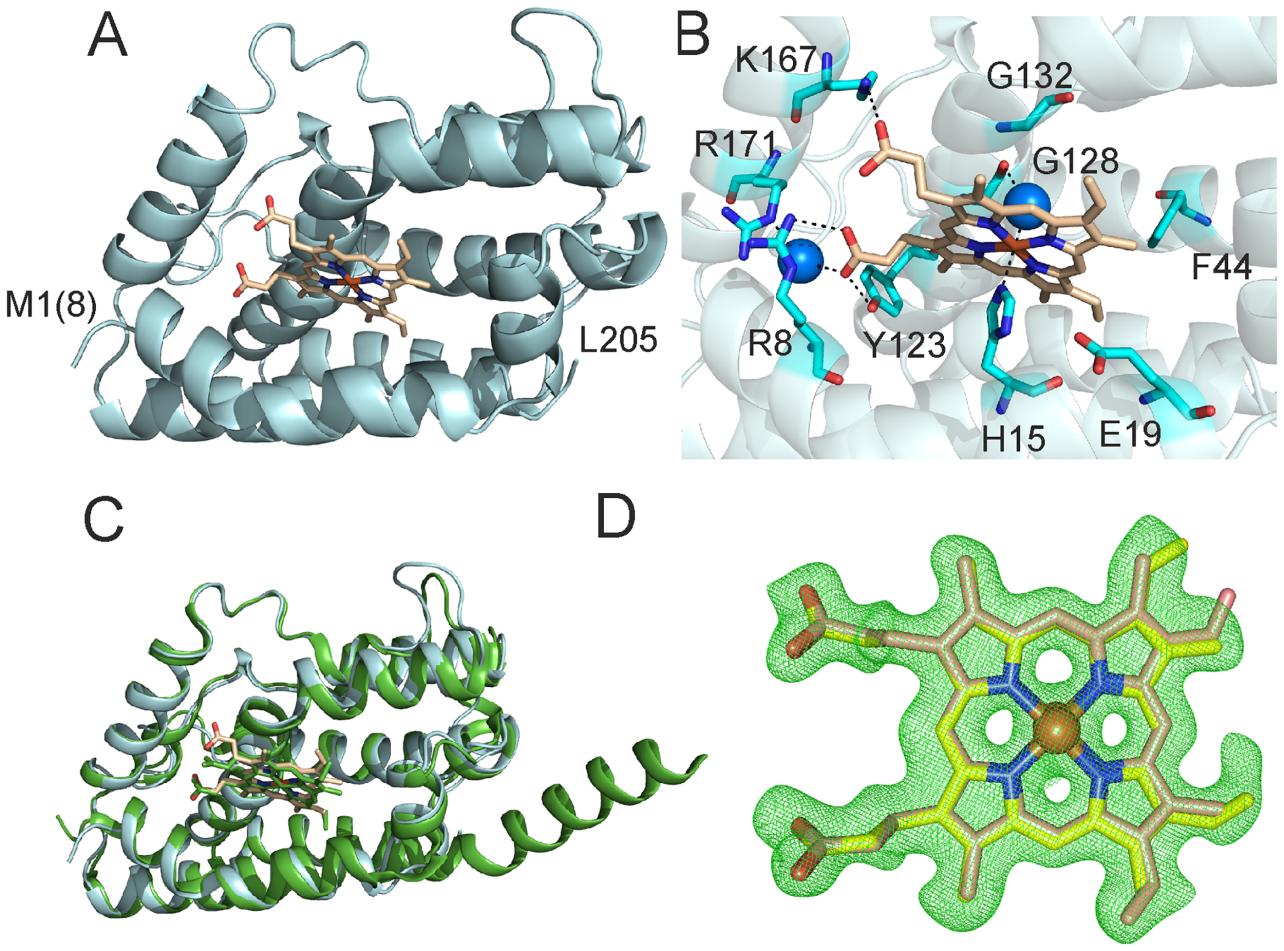
Crystallographic structure of the ferric heme LepHO complex. A) Cartoon representation of the LepHO-C26S-stop structure. B) Arrangement of the most relevant amino acid residues involved in heme binding to LepHO. C) Overall structural overlay of LepHO (cyan) with the HO-1 (PDB ID: **1WE1**, green) from *Synechocystis* sp. PCC6803. D) Final m*F*_o_−D*F*_c_ electron density map (green mesh) around heme ligand contoured at the 3σ level. The two orientations of the heme are displayed in yellow and light brown stick, respectively with oxygen atoms in red, and nitrogen atoms in blue. The Fe atoms are represented as orange spheres. Water molecules are depicted as blue spheres, and the heme ligand is represented in sticks. The amino acid residues in the recombinant LepHO are numbered according to the sequence of the wild type enzyme. The methionine in position 8 (numbers in parenthesis) of the recombinant protein has been labeled as 1, and so on for the subsequent amino acids. The recombinant protein contains an extra amino-terminal sequence (GHMASGS) which has remained from the construct for expression and purification of the protein.

The folding of LepHO is similar to that of other known HOs; it consists of a single domain of approximately 50 x 40 x 35 Å composed mostly of α-helices (Fig 1A). The heme is sandwiched between the proximal and distal helices, and the heme-iron is axially coordinated by the Nε2 nitrogen atom of H15, which resides in the proximal helix and by a water molecule in the distal site (Fig 1B). Bond distances between the iron atom and its axial ligands are 2.2 Å, consistent with corresponding distances in other HOs. It has been reported for several HOs that the proximal ligand (H25 in the mammalian enzyme) is stabilized by the interaction with the carboxylic side chain of a glutamic acid residue (E29 in the mammalian enzyme) [26,34–36]. A similar stabilization is not observed in LepHO, where the Nδ1 atom of H15 is 3.5 Å away from the carboxyl group of E19. Comparison of the LepHO structure with HO-1 from *Synechocystis* sp. [26], the closest homologue with available structural information, showed a high degree of similarity. Superimposing the structures of both enzymes resulted in the alignment of 1134 atoms with root-mean-square (rms) deviation of 0.692 Å (Fig 1C). In the LepHO structure the heme β-*meso*, γ-*meso* carbon and one of the propionate groups are exposed to the aqueous environment, while the α- and δ-*meso* carbons are buried in the interior of the heme binding pocket (Fig 1A and 1B).

**Fig 2:**
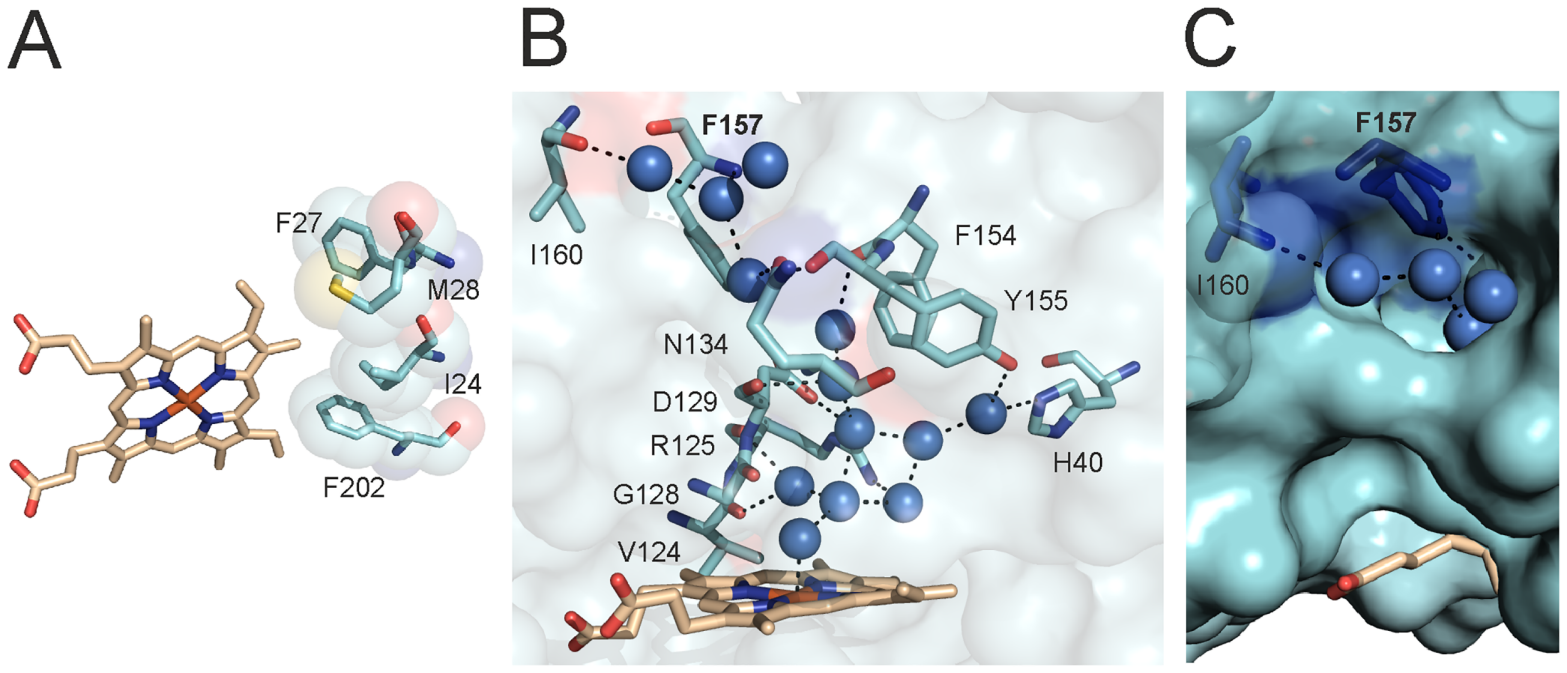
LepHO heme binding pocket and hydrogen bond network. A) Distinctive hydrophobic residues facing the α-meso carbon atom of heme in LepHO. B) LepHO residues, water molecules (blue spheres) and coordination bonds (broken lines) involved in the hydrogen bond distal site network. C) Detailed view of a LepHO structure in surface representation, where it can be seen that the hydrogen bonded network of structural waters reaches the surface of the protein and suggests a possible proton entry site.

We have previously used ^1^H NMR spectroscopy to determine that the heme seating in LepHO places the α-meso carbon where it is susceptible to oxidation that leads to the formation of α-biliverdin, and demonstrated with the aid of HPLC that the product of heme degradation is indeed α-biliverdin [18]. Moreover, the NMR data is consistent with the coexistence of two heme isomers that differ by a 180° rotation about the α-γ-meso axis. Note that heme rotation about this axis does not exchange the position of the α-meso carbon, which is consistent with the fact that α-biliverdin is the only product of heme degradation [18]. Analysis of the electron density in our crystallographic structure allowed us to corroborate the heme seating predicted from the ^1^H NMR analysis, as well as the presence of both heme isomers in LepHO (Fig 1D). Also, the structure of LepHO provides additional insights regarding the interactions between heme substituents and residues lining the heme pocket: Ionic and H-bonding interactions between the heme propionate groups and nearby side chains facilitate placement of the α-*meso* carbon atom where it can be hydroxylated (Fig 1B). The conserved R8 and K167 residues are 3.0 and 2.9 Å from the heme propionates, respectively. R171 contacts one of the heme propionates through a water molecule and Y123 can also H-bond with a heme propionate (2.6 Å). The heme α-*meso* carbon atom faces a hydrophobic wall formed by I24, F27, M28 and F202 (Fig 2A).

Differences among the heme binding cavity of LepHO and homologous enzymes were detected using the POCASA (V 1.1) software [37] (S3 Table). An indication of the distinct characteristics of the heme pocket in LepHO was also obtained analyzing the binding of known HO inhibitors. Incubation of LepHO with NaN_3_ and NaF at high concentration (10 mM) did not produce the expected low spin shift of the Soret band in the optical spectrum, indicating no coordination to the ferric heme iron. Similarly, NaCN induced a low-spin spectral shift, but it was necessary to incubate the protein in high concentration of the effector (50 mM) to reach saturation. Moreover, the CN-LepHO complex was unstable after removal of the excess of NaCN.

A signature of the distal pocket of HOs is the presence of two conserved glycine residues, which are thought to hydrogen bond with ligands bound to the heme-iron. Both glycine residues are postulated to be critical determinants of the catalytic cycle [14,26,38–40]. In LepHO, G128 is within hydrogen bond distance (2.85 Å) of a water molecule in the distal site near the heme. The amide nitrogen atom from the conserved G132 is at a greater distance (4 Å) from the same water molecule than the equivalent residues in other HOs (S5 Fig). In LepHO, G132 probably contributes mainly to the bending of the distal helix characteristic of all HOs. A relatively rigid network of water molecules, which extends from the active site to the surface of the enzyme, is also present in the crystal structure of LepHO (Fig 2B and 2C). This network consists of 13 water molecules, not counting the distal water ligand, and involves residues H40, V124, R125, G128, D129, N134, F154 and Y155. The water molecule that acts as axial ligand is linked by hydrogen bonding to two other water molecules, which in turn are stabilized by D129 and R125 (Fig 2B). This suggests that in LepHO both residues may be necessary for the delivery of H^+^ required for heme degradation. H40 in LepHO hydrogen bonds a water molecule in the network, a function that is usually carried out by an aspartate in other HOs. In fact, the H-bonding role played by the H40 residue is unique to LepHO since no other HO structure solved to date (S3 Fig) shows histidine at this position. Also, alignment of different HO sequences showed that H40 is only present in enzymes from pathogenic leptospiras [18].

The LepHO structure also revealed a pore on the surface of the protein, where the network of hydrogen bonded waters meets the surface. The side chains of F157 and the carbonyl of I160 point toward the interior of the opening and contribute to shape an entry to the network of hydrogen bonded waters leading to the heme active site (Fig 2B and 2C). Both residues are found in identical positions in all HOs from mammals, *Synechocystis* sp. and *C*. *diphtheria* [14,26,40], lining the depression on the protein surface that leads to the network of hydrogen bonded waters (Fig 2C). In addition, F157 belongs to a highly conserved group of phenylalanines and tyrosines found in all HOs that published evidence has pointed as essential for catalysis [41]. These observations motivated us to analyze the potential contribution of F157 to the integrity of the network of hydrogen bonded waters and to the catalytic activity of LepHO. Results from these experiments are presented below.

### Analysis of the role of F157 by site-directed mutagenesis

**Fig 3:**
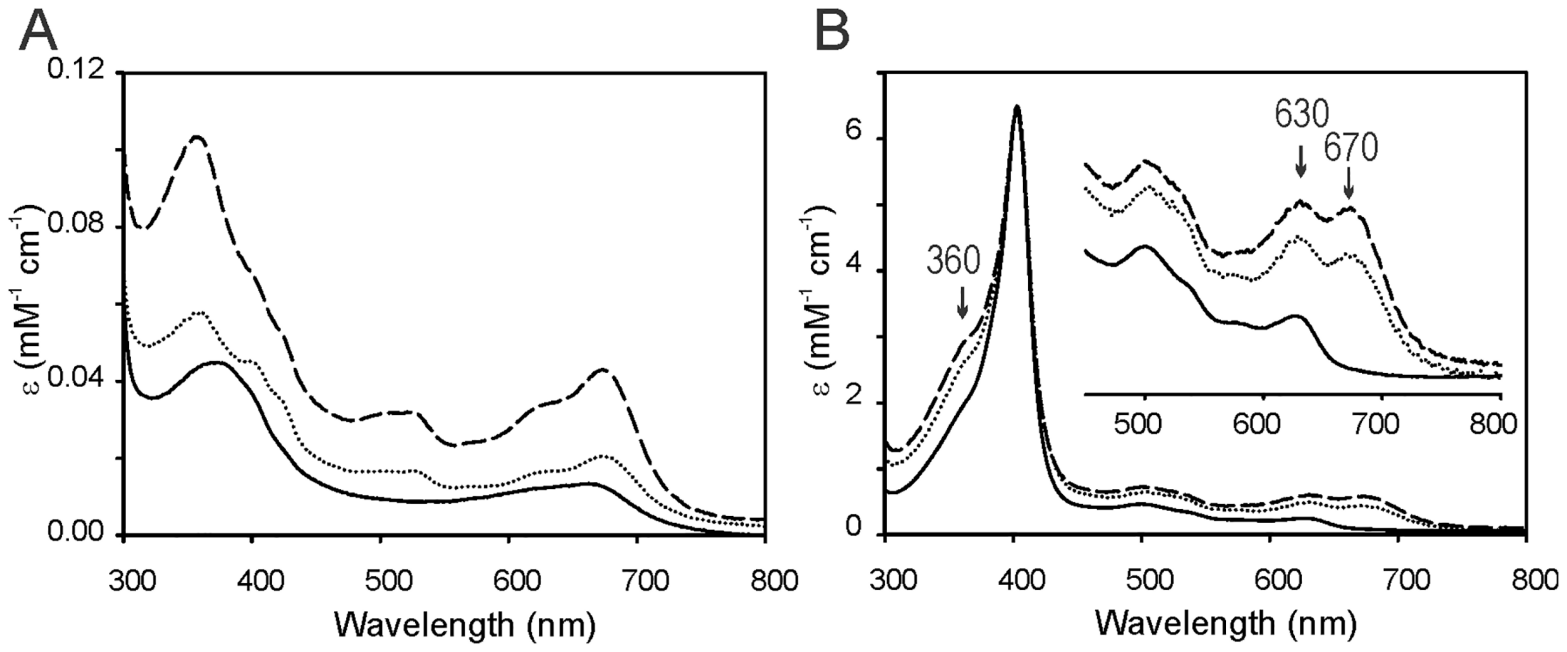
Optical absorption spectra of the purified wild type LepHO, F157I and F157A variants. Spectra of the different LepHO variants purified by metal affinity chromatography before (A) and after (B) ferric heme complex formation: wild type LepHO (―); F157A (…) and F157I (---).

To study the role played by F157 in enzyme function, stability and catalysis, this residue was mutated to isoleucine or alanine. The wild type and F157 mutants were expressed and purified as active soluble proteins. It has been previously reported that *E*. *coli* cells expressing HOs turn bright green due to the accumulation of biliverdin, the final product of the catalytic turnover of the enzyme [42]. Overexpression of wild type LepHO in *E*. *coli* also causes accumulation of biliverdin and imparts the *E*. *coli* cells with the corresponding bright green color. In contrast, overexpression of the LepHO F157 variants causes the *E*. *coli* cells to turn dark green color, which is clearly different from the bright green color observed when wild type LepHO is expressed. The distinct color observed in *E*. *coli* cells overexpressing the F157 mutant provided the first indication that the LepHO variant does not produce the same product as the wild type enzyme during recombinant expression. Comparison of the electronic absorption spectra of purified wild type and mutant proteins reveals significant differences (Fig 3A).

The F157I mutant displays bands at 357, 525 and 678 nm, which are reminiscent of the absorption spectrum reported for the HO-verdoheme complex (λ_max_: 352, 404, 540 and 678 nm) [43–45]. The F157A variant showed similar spectral characteristics, albeit with less defined absorption bands at 357 and 678 nm. These spectral properties are clearly different from those exhibited by the purified wild type protein, which shows a broad band near 670 nm, characteristic of biliverdin. Titration of the wild type and F157 mutants with hemin resulted in changes of the electronic absorption spectra that are consistent with the formation of a ferric heme complex. Upon removal of excess heme, the wild type enzyme displays a characteristic Soret maximum at 403 nm and a charge-transfer band at 630 nm, indicative of high spin. In comparison, the spectra of the F157 mutants, in addition to the Soret band and high spin marker band, also present an absorption band *ca*. 360 nm and peaks at 630 and 670 nm (Fig 3B).

### Catalytic turnover of the ferric heme complex

**Fig 4:**
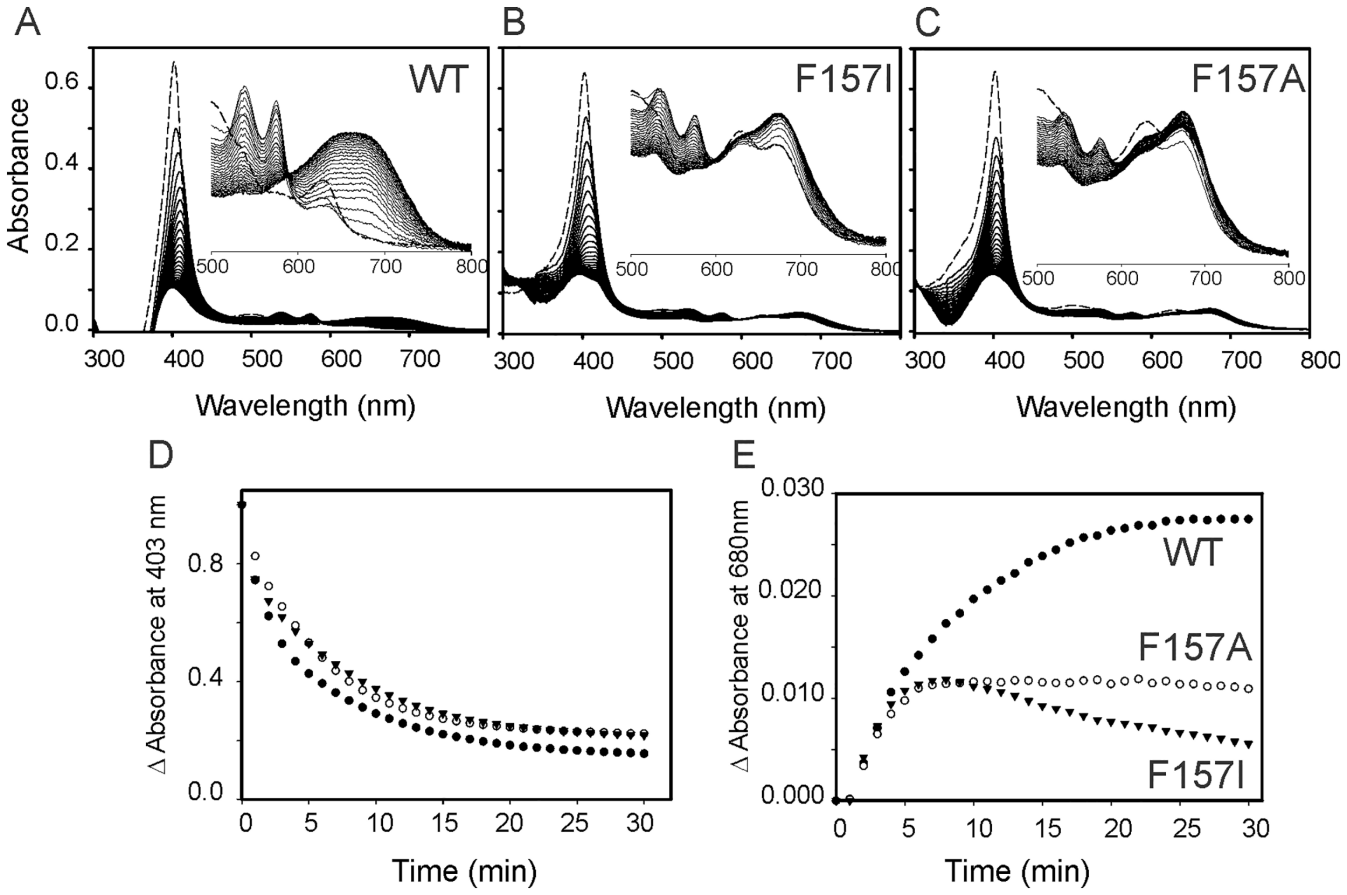
Absorption spectral changes of the LepHO-heme complex during the NADPH/LepFNR-supported heme degradation. Time dependent absorption spectra of wild type LepHO (A), F157I (B) and F157A (C), before (---) and after (―) the addition of LepFNR and NADPH: Experimental conditions are as indicated in Materials and methods. The inset shows an enlargement of the spectral region between 500 and 800 nm. The time-dependent decay of the intensity at 403 nm (D) and the increase at 680 nm (E) were obtained from the spectra shown in panels (A) to (C). Wild type LepHO (●); F157I (▼) and F157A (○).

We first examined the heme degrading activity of LepHO employing the NADPH/LepFNR system as electron donor. The spectral changes obtained with the wild type protein are shown in Fig 4A.

Immediately after addition of the reductase, the ferric heme complex was reduced, and the formation of the oxyferrous complex was detectable by the decrease in intensity and shift of the Soret band from 403 to 410 nm with the concomitant appearance of α/β peaks at 538 and 575 nm, respectively. As the bands characteristic of the oxyferrous complex decrease in intensity and then disappear, a new broad peak centered at 680 nm appears, signaling the accumulation of biliverdin. Results from similar experiments carried out with the F157 mutants are shown in Fig 4B and 4C. Accumulation of the oxyferrous complex is lower than in the wild type enzyme, as indicated by the modest shift of the Soret peak and relatively low intensity of α/β bands. As the reaction proceeds, the peaks characteristic of the oxyferrous complex disappear, but the broad band at 680 nm characteristic of biliverdin is not formed. Instead, a peak at ca. 670 nm is formed with the concomitant appearance of an absorption band at 525 nm. The spectrum obtained after 30 min of reaction is similar to those reported for HO-verdoheme complexes [46], suggesting that verdoheme is the final product of heme degradation by the F157 mutants of LepHO.

The reduction of heme and formation of the oxyferrous complex is practically unaffected (Fig 4D), indicating that the mutations do not alter the capability of LepHO to interact with LepFNR and accept electrons. However, the production of biliverdin by the mutant enzymes is not observed, showing an alteration in the heme degradation process (Fig 4E). To establish whether biliverdin or verdoheme was present in the reaction mixtures a mass spectrometry positive-ion mode analysis was performed. Dyes extracted from the solution after the heme degradation reaction carried out by wild type LepHO showed a single high abundance peak with a mass-to-charge ratio (m/q) 583.25 (data not shown). This value is identical to the positive ion of an authentic biliverdin standard. Significantly, the peak at m/q = 583.25 was absent from the extract of the solution obtained upon heme degradation carried out with the F157I variant. Additional evidence corroborating that the F157I variant degrades heme to verdoheme was obtained by the analysis of the electronic absorption spectrum of the pigments extracted with pyridine and chloroform from the reaction products (S6A Fig). This spectrum bears a close resemblance to that reported for verdoheme in the same solvent [44]. When this compound was analyzed by HPLC using a Hypersil ODS C18 column, it eluted at 31.8 min while the reaction product of wild type LepHO eluted at 15.1 min as well as a biliverdin standard (S6B Fig). Taken together, the data demonstrate that heme turnover in the F157I mutant is arrested at the verdoheme stage.

The heme degrading activity of the F157I mutant was measured using the NADPH/LepFNR system as electron donor and the data used to calculate and compare the apparent LepFNR Michaelis constants with corresponding values obtained from the wild type enzyme. A V_max app_ of 0.086 ± 0.003 μmol F157I reduced min^−1^.mg LepFNR^−1^ and, a K_m app_ of 2.71 ± 0.31 μM were obtained. The parameters estimated using the wild type LepHO measured under identical conditions were: V_max app_ = 0.177 ± 0.007 μmol LepHO reduced min^−1^.mg LepFNR^−1^ and K_m app_ = 1.77 ± 0.21 μM) [18]. From these data, it is evident that heme degrading activity is considerably altered in the mutant enzyme.

**Fig 5:**
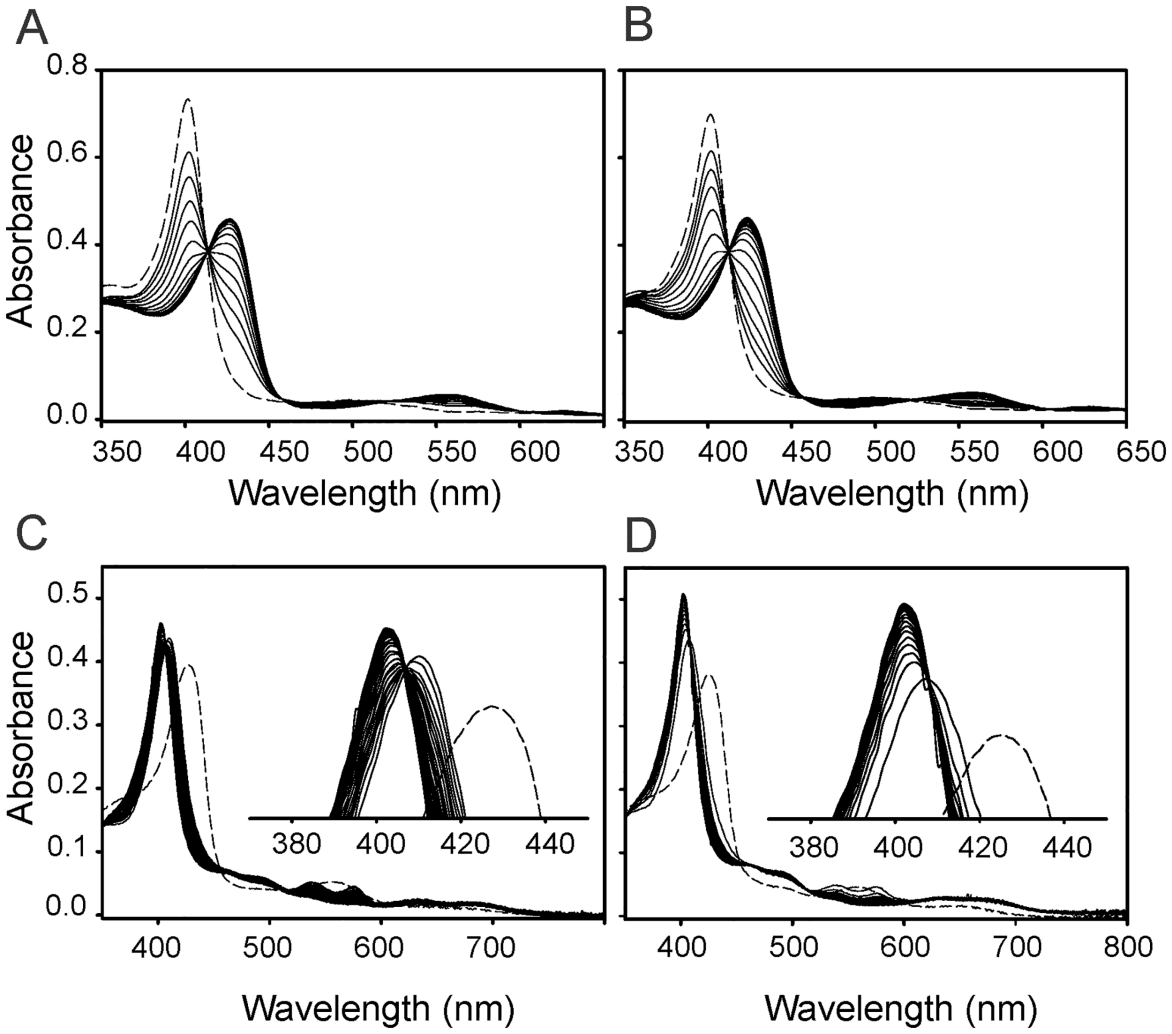
Reduction of the ferric LepHO-heme complex in anaerobic conditions and subsequent autooxidation in air. Absorption spectral changes of 6 μM wild-type LepHO (A) or F157I mutant (B) before (---) and after the addition of 1.2 μM LepFNR (―) in the presence of NADPH. Autoxidation of the ferrous LepHO-heme complex in air. Wild type LepHO (C) and F157I mutant (D) before (---) and after bubbling with O_2_ (―).

Similar observations were obtained when the heme degrading activity of LepHO was carried out with ascorbate as electron donor. The addition of 5 mM ascorbic acid to the wild type protein resulted in a gradual decrease of Soret band intensity and the appearance of a broad absorption peak centered near 680 nm, indicating that wild type LepHO is able to use ascorbate to support the degradation of heme to biliverdin (S7A Fig). In contrast, and as observed in experiments in the presence of NADPH/LepFNR, the same spectral changes did not occur when the F157 mutants were reacted with an identical amount of ascorbate (S7B and S7C Fig). Instead, a peak ca. 670 nm, like that observed in the presence of NADPH/LepFNR, indicates that the product of the coupled oxidation carried out by the F157I mutant is also arrested at the verdoheme stage. To further evaluate the reactivity of the F157I variant, the electron transfer reaction from reduced LepFNR to LepHO was examined by optical absorption spectroscopy. Reduced LepFNR was prepared in a glovebox by the addition of one equivalent of NADPH to the oxidized reductase. The solution containing reduced LepFNR was added anaerobically to the LepHO-heme complex (5:1 LepFNR:LepHO ratio). Formation of the ferrous complex was monitored by absorption spectroscopy following the appearance and growth of bands at 426 and 562 nm. The spectral changes observed with wild type LepHO were similar to those observed with the F157I mutant (Fig 5).

**Fig 6:**
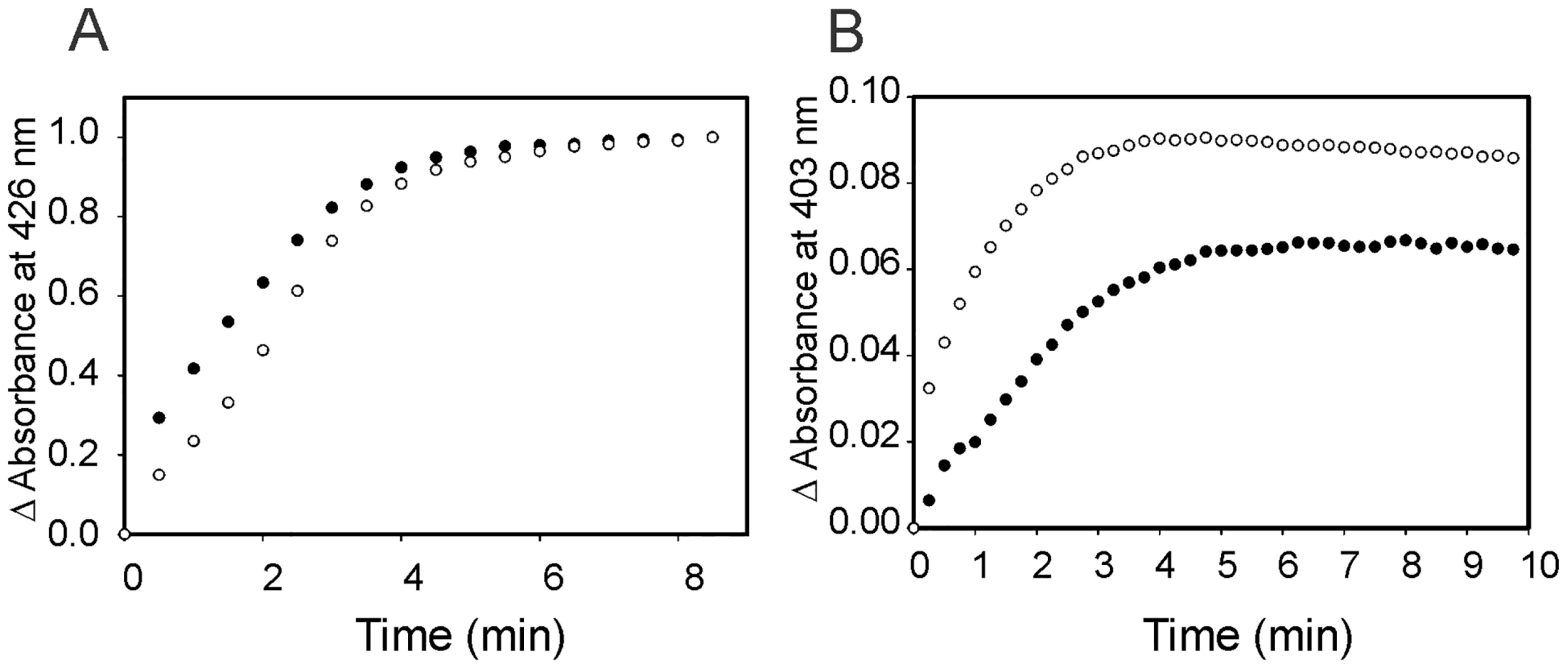
Reduction of the ferric LepHO-heme complex in anaerobic conditions and spontaneous reoxidation. Time dependent formation (A) and autoxidation (B) of the ferrous heme complex of wild type LepHO (●) and F157I mutant (○) as monitored by variations in absorbance at 426 and 403 nm, respectively. Data extracted from the spectra shown in Fig 5.

Changes in absorbance at 426 nm plotted as a function of time showed no significant difference between the two proteins. When similar experiments were performed with an excess of NADPH to mimic the conditions of the heme turnover assay described aerobically, identical results were achieved. Taken together, these observations indicate that delivery of the first electron required to reduce resting state LepHO is not affected by the F157I mutation (Fig 6A).

We also investigated the susceptibility of the corresponding oxyferrous complexes to autoxidation. To this end, a solution containing the ferrous LepHO-heme complex was removed from the glovebox, bubbled with O_2_, and monitored by recording spectral changes. During autoxidation of the ferrous complex, the intensity of the 426 and 562 nm peaks decreased and the formation of the ferric complex was evident by the shift of the Soret band to 403 nm and the disappearance of α/β peaks at 538 and 575 nm. In contrast to the stability displayed by the oxyferrous complex of wild type LepHO, the oxyferrous complex of the mutant autoxidized relatively fast. Hence, the low-level accumulation of the oxyferrous complex during heme degradation, as judged by the low intensity of the corresponding absorption bands (see Fig 4B), and the faster autoxidation of the oxyferrous complex (Fig 6B), suggest that the instability of the latter in the F157I mutant is lower than in the wild type enzyme.

### The reaction of LepHO with H_2_O_2_

**Fig 7:**
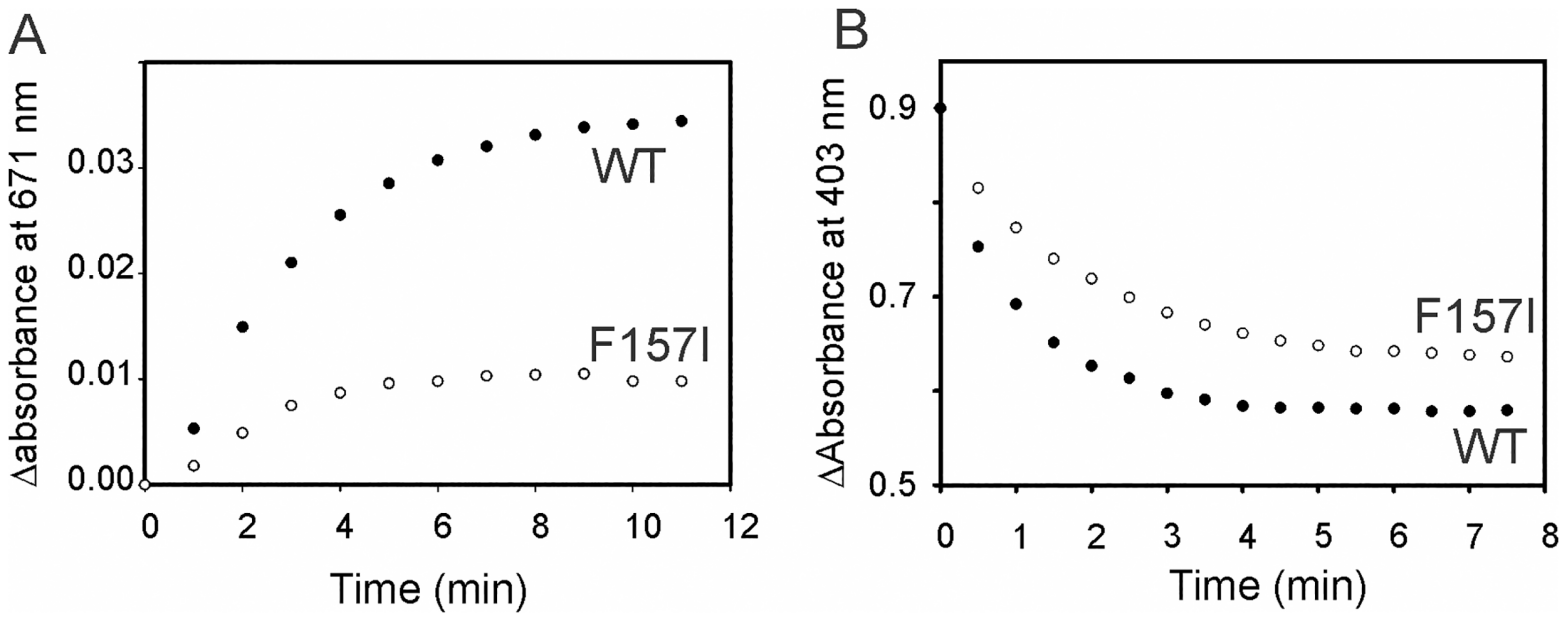
Conversion of ferric LepHO-heme complex to verdoheme by addition of H_2_O_2_. Time dependent heme hydroxylation of wild type LepHO (●) and F157I mutant (○) by H_2_O_2_ in aerobic conditions as monitored by increase in absorbance at 671 nm (A) or under anaerobiosis as monitored by decrease in absorbance at 403 nm.

The efficiency of heme hydroxylation was analyzed by assessing the reaction between the ferric heme complex and H_2_O_2_. Upon addition of H_2_O_2_ to the resting state, HO is known to form a ferric hydroperoxide intermediate (Fe^III^-OOH), which reacts with heme to give *meso*-hydroxyheme. In the presence of O_2_ the *meso*-hydroxyheme is rapidly converted to verdoheme with the simultaneous elimination of the *meso*-carbon atom as CO [47,48]. Formation of verdoheme can be easily observed by an absorption band ca. 671 nm. Hence, as expected, addition of one equivalent of H_2_O_2_ converted the heme bound to wild type LepHO into verdoheme. In contrast, addition of one equivalent of H_2_O_2_ to resting state F157I results in significantly slower formation of verdoheme and in much lower accumulation (Fig 7A).

This observation is significant because it suggests that the mutation affects the efficiency of meso carbon hydroxylation by the Fe^III^-OOH intermediate, and possibly the subsequent conversion of meso-hydroxyheme to verdoheme. To gain additional insight, the reaction between resting state enzyme and H_2_O_2_ was investigated in the absence of O_2_: Addition of H_2_O_2_ to resting state wild type LepHO causes the Soret band at 403 nm to become broader and to decrease in intensity, with a concomitant slight increase in the absorbance between 640–690 nm. These changes are in agreement with the formation of the *meso*-hydroxyheme complex, as have been reported for other HOs [49,50]. In comparison, the reaction carried out by the F157I mutant is slower than that carried out by the wild type LepHO (Fig 7B).

When all the above-described observations are taken together, the picture that emerges indicates that the F157I mutation affects the reactivity of LepHO by making the oxyferrous complex less stable, which possibly lowers the efficiency by which the Fe^III^-OOH intermediate forms. In addition, the lower reactivity of the resting state F157 mutant toward H_2_O_2_ under aerobic and anaerobic conditions suggest that the conversion of the Fe^III^-OOH intermediate to meso-hydrohyheme, and the conversion of the latter to verdoheme, are both adversely affected. We propose that these effects are related to disruption of the hydrogen bonding network communicating the surface of the protein, where F157 lines the entry port to the network, and the heme active site.

### Putative model of the LepHO-LepFNR complex during catalysis

**Fig 8:**
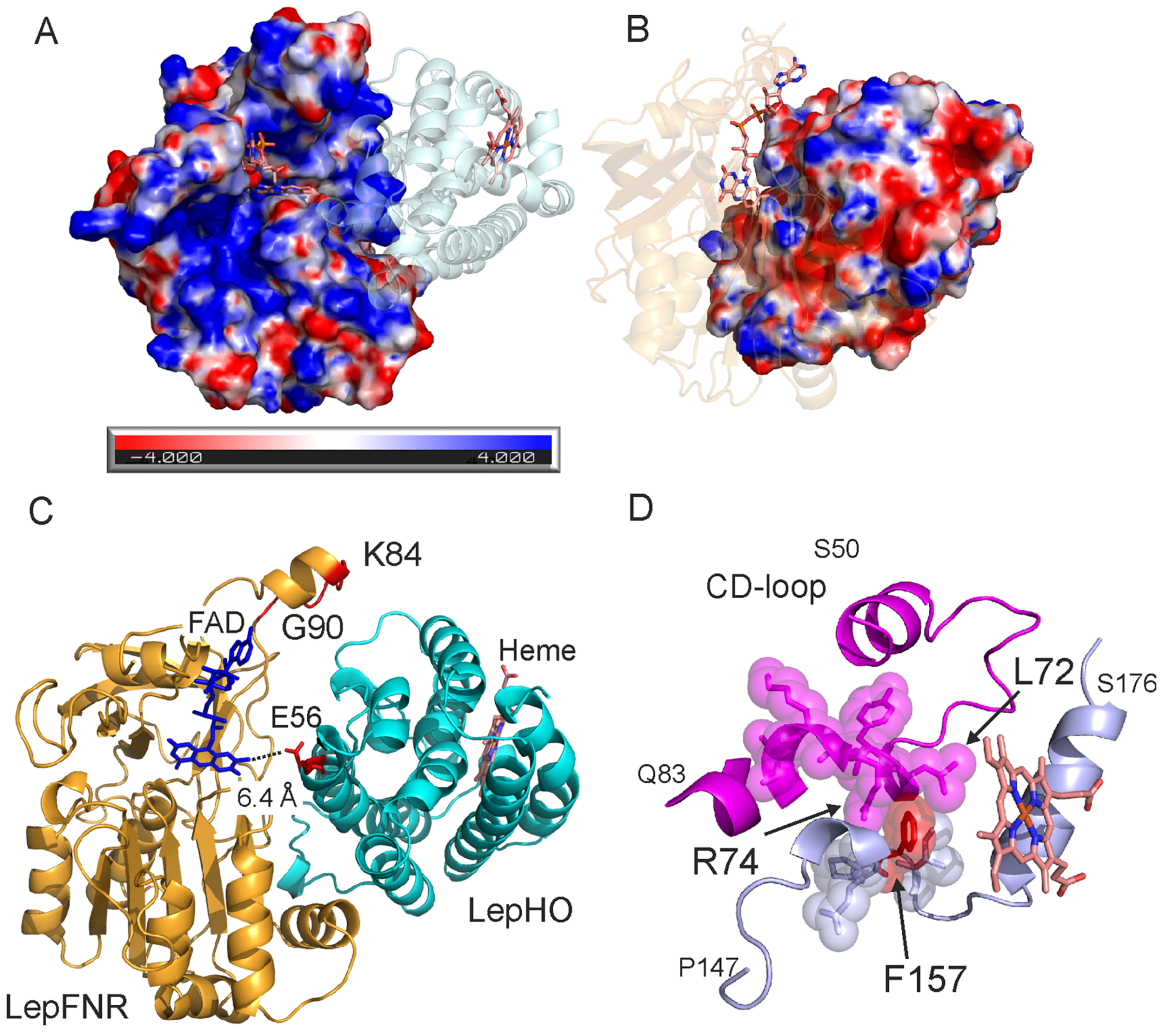
Models of the complex between LepHO and LepFNR and distal modulation of the LepHO activity. Electrostatic surface potential of LepFNR (A) and LepHO (B) as obtained by using the PDB2PQR service [51], the APBS plugin [52] and Pymol 1.8. The color scale was set from -4 kT/e (red) to +4 kT/e (blue). Proteins were slightly rotated to show the surfaces involved in complex formation. Putative model of the complex LepHO-LepFNR (C) obtained with ClusPro 2.0 [53]. The CD-loop homologous to that found in human HO-1 [54] is depicted in magenta (D). The region containing F157 (red) is presented in light blue and heme in light brown.

Crosslinking experiments of LepFNR and LepHO in solution under catalytic conditions allowed us to observe the existence of a physical interaction between both proteins (S8 Fig). Moreover, we detected that the complex is formed with an apparent 1:1 stoichiometry. To further investigate the association of these two proteins, the electrostatic surface potentials of LepHO and LepFNR were calculated. In LepHO, the proximal region of the heme binding pocket is positively charged, while the distal region is negatively charged. The surface of LepFNR displays positive potential adjacent to the cavity where the ribityl atoms of FAD bind, which is the region, expected to interact with its protein partners (Fig 8A and 8B).

A set of probable complexes was calculated using the 2.0 ClusPro software [53]. A model in which the electrostatic surfaces of LepHO and LepFNR are complementary and electron transfer from the isoalloxazine moiety would be feasible was obtained (Fig 8C). In this model, the residues N14, K17, K84, L86, K156, E279, K280, E298, K300 and G305 in LepFNR, and E49, E53, R57, E71, N76, E80, N95, K109, E112 and N161 in LepHO might participate at the complex interface. The FAD is appropriately oriented for the electronic transfer to the nearest amino acid of LepHO E56, which is at 6.4 Å (Fig 8C). Therefore, the main region of interaction on the LepHO surface may occur on an opposite site from the heme binding pocket. A distinguishing feature of the LepFNR structure is a small helix of the loop G76 –Y91 [55]. The sequence from K84 to G90 which belongs to this small helix, absent in all other bacterial and plant FNRs, is facing LepHO in our model. It has been pointed that this region may have low mobility on the basis B-factor analyses [55]. We propose that this exposed structure would be necessary for the interaction between LepFNR and LepHO and for a productive orientation of the catalytic complex.

To provide support for our model, we prepared a LepFNR mutant enzyme in which the loop region between K84 and G90 was deleted (Fig 8C). Thus, a coding sequence for a LepFNRΔK84-G90 was constructed, expressed and the mutant enzyme purified. The mutant LepFNR displayed a UV-Vis spectrum almost identical to that of the wild type enzyme (S9A Fig). Moreover, the kinetic parameters for the NADPH diaphorase activity of the variant (K_m_ 18 ± 2 μM, *k*_cat_ 257 ± 9 s^-1^) showed to be very similar to those measured for the wild type enzyme (K_m_ 22 ± 1 μM, *k*_cat_ 280 ± 4 s^-1^). These observations indicate that the protein is properly folded and that the catalytic processes involving the nucleotide and small artificial substrates remain unchanged. In contrast, the heme degradation activity by LepHO using this mutant enzyme and NADPH as electron donor showed a slower decrease of the Soret band at 403 nm and altered increase of the absorbance at 680 nm, achieving only 60% of the total conversion catalyzed by the wild type enzyme (S9B and S9C Fig). Consequently, we suggest that this experimental evidence supports the proposed model of LepFNR-LepHO interaction.

## Discussion

LepHO, which carries out the degradation of heme to biliverdin and free iron receives the electrons required for the catalytic reaction from a plastidic (plant)-type ferredoxin-NADP^+^ reductase also found in *Leptospira interrogans* [18]. Hence, the catalytic heme degradation supported by LepHO and its cognate electron transfer partner enables *L*. *interrogans* cells to utilize iron from host heme and provide a mechanism for circumventing the iron-limitation conditions imposed by the host defense system. The aim of this investigation was to determine the structure of LepHO and use the structural insights to probe the effect of perturbing surface residues at the putative entrance to the network of hydrogen bonded waters that connect the heme active site and the interface between the protein and the aqueous environment.

### The LepHO structure

Modeling the LepHO structure *in silico* revealed the existence of an extended, 20-residues long C-terminal region of the enzyme, which we surmised is disordered and may have stymied our prior attempts at growing single crystals. We capitalized from the *in silico* findings by designing a variant where the last 20 residues were removed, and the unique cysteine (C26), which the model revealed is on the protein surface, was replaced by a serine. The variant enzyme, which we termed LepHO-C26-stop, is functionally equivalent to the wild type LepHO *in vitro*. LepHO-C26-stop was crystallized and the structure of its ferric heme complex solved. Comparison of the structure with other crystallized HOs shows that the LepHO enzyme shares the same overall fold and structural conservation of most functionally relevant residues. Certain features of LepHO are of note. The heme seating in the LepHO structure is consistent with that seen in all HO enzymes with the exception of the heme oxygenase from *Pseudomonas aeruginosa*, which places the β-meso (or δ-meso) carbon where it is hydroxylated by the Fe^III^-OOH intermediate [56]. The m*F*_o_−D*F*_c_ electron density omit map around the hemin ligand contoured at the 3σ level indicates the presence of two LepHO populations with different heme orientation, displaying a 180° rotation about the α-γ-meso axis. We reported previously that the presence of two heme orientations was evident in the 1H NMR spectrum [18]. This behavior on the hemin molecule is not novel, as it was previously reported in other HOs [38,57,58] and in the highly-related enzyme, the hemophore HasA from *P*. *aeruginosa* [59]. The functional relevance of heme disorder in most cases is negligible, as this is probably the case in LepHO, which produces alpha-biliverdin regardless the presence of the two heme positioning, as we have previously observed [18]. LepHO oxidizes heme to α-biliverdin, a finding that is in agreement with our previous interpretation of the ^1^H NMR spectrum of cyanide inhibited-enzyme [18]. In LepHO, the distance between G128 and the oxygen atom bound to the heme iron (2.85 Å) is in agreement with the establishment of an interaction. On the other hand, G132 is 4 Å away from the distal ligand. However, considering that the distal helix is capable of adopting different conformations and that minimal changes would put G132 closer to the heme, we cannot exclude its direct participation in some steps of the catalytic mechanism, as was previously reported for the homologous residues G139 and G143 in human HO-1 [7,13].

### Replacing F157 at the protein surface affects catalytic activity, probably by disrupting the H-bonded network of structural waters

The structures of HO enzymes possess a network of hydrogen bonded structural waters that extends from the catalytic site to the surface of the enzyme. Analysis of the LepHO structure revealed that residues F157 and I160 are part of a depression on the enzyme surface which likely constitutes the outermost section lining the network of hydrogen bonded waters, or said in other words, the probable entrance to the network from the aqueous environment. F157, which is conserved in all HOs, is located 16.5 Å from the heme binding site. The hydrogen bonded network is crucial for HO catalytic activity, and prior reports have shown that mutating residues in the network close to the active site affect the catalytic activity. Consequently, we decided to study the effect of mutating F157. We found that a striking consequence of replacing F157 is the inability of the mutant enzymes to carry out the complete degradation of heme to biliverdin; rather, the oxidation of heme is arrested at the verdoheme stage. Additional probing indicated that the oxyferrous complex in the F157 variants is less stable (faster autoxidation) than that of the wild type enzyme. Moreover, experiments in which the enzymes were reacted with H_2_O_2_ under aerobic and anaerobic conditions strongly suggest that heme hydroxylation by the Fe^III^-OOH intermediate and subsequent conversion of meso-hydroxyheme to verdoheme occur in the mutant with significant less efficiently than in the wild type enzyme. In aggregate, these observations indicate that mutating the surface-located F157 significantly alters several steps of the reaction mechanism, and suggest that a reasonable explanation for the observations may be the disruption of the hydrogen-bonded network of structural water. This disruption can be a consequence of subtle structural and/or dynamical perturbations induced by the mutations, an interpretation that agrees with previous work showing that either subtle structural changes [7,13,54] or dynamical changes [6] of the H-bonded network have an adverse effect on catalytic activity.

Recently, a distal regulation of the heme binding of the human HO-1 by conformational fluctuations was proposed [54]. The authors solved the structure and analyzed the conformational dynamics of the enzyme using NMR. They found that a surface loop (CD-loop, Fig 8D), distal to the catalytic site, participates in the regulation of the heme binding throughout intramolecular fluctuations. The CD-loop is found, with a lower level of similarity but with identical position than in human HO-1, in the crystal structure of LepHO and extends from S50 to Q83 residues. This loop is in close contact with an unstructured region that spans from P147 to N164 and with a helix that starts at G165 and ends at S176. The F157 residue is sandwiched between L72 and R74 of the CD-loop (Fig 8D). The F157, L72, and R74 residues show a high degree of conservation among bacterial and eukaryotic HOs [18]. It can be reasonably inferred that any alteration in the interaction, movement or change in F157 is to be transmitted along the protein structure, generating a variation of the catalytic performance thereof. Moreover, this region is involved, according to our model, in the interaction with the redox partner LepFNR.

### Proposed model for the productive complex LepFNR-LepHO

Since the structure of the reductase LepFNR has been reported [55], the structure of LepHO provides the opportunity to begin investigations aimed at determining the structure of the cognate LepHO-LepFNR complex, in order to gain additional fundamental understanding regarding electron transfer between these two proteins. The interaction between LepFNR and LepHO is likely to occur transiently and, as in the case of other FNR-protein substrate complexes [60], is probably driven by electrostatic interactions. Analysis of the protein structures together with the results obtained from cross-linking experiments allow us to propose the first model for the interaction between a ferredoxin-NADP^+^ reductase and a heme oxygenase. The surface of LepFNR exhibits a vast positively charged area near the FAD where the isoalloxazine moiety is likely to deliver the electrons to LepHO (Fig 8A). Accordingly, the model proposes that the negatively charged regions on the surface of LepHO may help steer the proteins toward productive interactions. Among the residues thought to be involved in this interaction, the conserved E102 has been implicated in the interaction of HO-2 with its redox partner cytochrome P450 reductase. Cytochrome P450 reductases are different enzymes than FNRs but their C-terminal dinucleotide-binding domains are similar [61]. Calculation of the surface electrostatic potential of the cytochrome P450 reductase reveals that the region near the FMN binding site has a strong negative charge. Consequently, primarily basic residues are necessary on the surface of HO-1 and HO-2 for the interaction with their redox partner, which is distinct from what is observed with the LepFNR-LepHO complex. Clearly, additional work is necessary to understand the complex in detail, as well as the implications in electron transfer and heme degradation activity. These studies are currently underway in our laboratories.

## Conclusions

In this work, we have solved the crystal structure of the *L*. *interrogans* heme oxygenase-heme complex at 1.73 Å resolution. The structure revealed residues involved in the hydrogen-bonded network of structural water molecules that extends from the heme distal ligand to near the protein surface in LepHO. The entrance to the hydrogen bonding network at the protein surface is revealed by a depression formed in part by residue F157. Replacement of F157 significantly alters the catalytic activity of LepHO and reveals long-range communication between the outer limits of the hydrogen-bonded network of structural waters and the heme active site. Experiments designed to probe distinct steps of the catalytic cycle showed that the F157I mutation destabilizes the binding of O_2_ to iron due to changes caused by the distal environment of the active site, either by disturbances generated in the network of water molecules or through the flexibility of the distal helix. These perturbations also affect adversely the efficiency of heme hydroxylation and its subsequent conversion to verdoheme. Hence, we showed that disruptions to the H-bonded network, even in places most remote form the active site, such as the protein surface, have a detrimental effect on heme oxidation activity. Finally, by analyzing the crystal structures of ferredoxin-NADP^+^ reductase and heme oxygenase, a preliminary model for the association of these proteins has been proposed.

## Supporting information

S1 Fig Mechanism of heme degradation by heme oxygenase. A) Proposed heme degradation pathway in HO. B) Characteristic absorption spectra of complexes formed during heme degradation catalyzed by LepHO. Arrows and numbers indicate wavelengths of characteristic absorption bands of the ferric (—, a), ferrous (···, b), oxyferrous (---, c), verdoheme (- ·· -, d) and biliverdin (- · -, e) complexes.
(PDF)

S2 Fig Predicted cartoon model of the LepHO structure. Two models were generated by homology with the I-TASSER program (Roy A, Kucukural A, Zhang Y. I-TASSER: a unified platform for automated protein structure and function prediction. Nat Protoc. 2010;5: 725–38. 10.1038/nprot.2010.5) using the *Synechocystis* sp. PCC6803 HO-1 (PDB ID: **1WE1**, green) and the *Synechocystis* sp. PCC6803 HO-2 (PDB ID: **1WO1**, yellow) as structural templates. A superimposed representation of both models is presented where C26 and E206 residues are depicted in purple.
(PDF)

S3 Fig Multiple sequence alignment of LepHO with close homologs. The relevant residues involved in heme binding (bold, red), mutated or removed for improving crystallizability (bold, green), proposed participating residues in the electron transfer path (bold, purple) and F157 studied in this work (bold, blue) have been highlighted.
(PDF)

S4 Fig Analysis of purified LepHO variant for crystallization. Electrophoresis in 12% polyacrylamide gels in the presence of SDS (A) or under native conditions (B) of the wild-type LepHO (lane 1) or LepHO-C26S-stop (lane 2). Protein standards (kDa) are shown in the first lane in (A). Time dependent absorbance changes at 403 nm (C) and 680 nm (D) were recorded for reactions containing 1 μM LepFNR, 300 μM NADPH, 0.1 mg/ml catalase and 6 μM of wild-type LepHO (●) and LepHO-C26S-stop (Δ) as ferric complexes. The decay of the absorbance at 403 nm indicates heme rupture while increase at 680 nm shows biliverdin formation.
(PDF)

S5 Fig Arrangement of the distal G128 and G132 residues in the LepHO structure. The water molecule located near heme is represented as a blue sphere. Distances are stated in Å and represented as dashed lines.
(PDF)

S6 Fig Identification of the reaction product of the heme degradation by the F157I mutant. Spectral characterization (A) and HPLC analysis (B). After catalytic conversion of heme by the enzyme, 5% (v/v) pyridine was added, the products extracted with chloroform and analyzed as described in Material and methods.
(PDF)

S7 Fig Catalytic turnover of heme by LepHO in the presence of ascorbate. Spectroscopic changes of wild-type LepHO (A), F157I (B) and F157A (C) mutant enzymes before (---) and after (―) addition of 5 mM ascorbic acid. The inset shows an enlargement of the region between 500 and 800 nm.
(PDF)

S8 Fig Analysis of the LepFNR-LepHO interaction by chemical crosslinking. SDS-PAGE (12%) (A) and western blots using Anti LepFNR (B) or Anti LepHO (C) antibodies. Crosslinking reactions were carried out for 30 min using 25 mM EGS in 20 μl of 25 mM HEPES-KOH, pH 7.5, containing LepHO-heme and/or, LepFNR (12.5 μM protein concentration each) as indicated on the figure. Numbers to the left indicate molecular weight markers in kDa. Proteins bands that appear by crosslinking of the complex LepHO-LepFNR (61 and 88 kDa) are indicated with an asterisk.
(PDF)

S9 Fig Evaluation of the spectroscopic characteristics of LepFNR and LepFNRΔK84-G90, and their capabilities to supporting heme degradation by LepHO. A) Absorption spectra of LepFNR (―) and LepFNRΔK84-G90 (···). Time dependent absorbance changes at 403 nm (B) and 680 nm (C) were recorded for reactions containing 6 μM LepHO, 300 μM NADPH, 0.1 mg/ml catalase and 1 μM of wild-type LepFNR (●) or LepFNRΔK84-G90 (▼). The decay of the absorbance at 403 nm indicates heme rupture while increase at 680 nm shows biliverdin formation.
(PDF)

S1 Table Nucleotide sequences of synthetic oligonucleotides used for the construction of the *L*. *interrogans* heme oxygenase and ferredoxin-NADP^+^ reductase mutants. (PDF)

S2 Table X-ray diffraction data collection and refinement statistics from the LepHO-C26S-stop ferric heme complex. (PDF)

S3 Table Volume and depth of heme oxygenase prosthetic group pockets from different organisms. (PDF)
