# Supplementary figures and images for: Structural and mutational analyses of the Leptospira interrogans virulence-related heme oxygenase provide insights into its catalytic mechanism
Source: PLoS One. 2017 Aug 3;12(8):e0182535. doi: 10.1371/journal.pone.0182535 (PMC5542595; doi:10.1371/journal.pone.0182535)

**S1 Fig. Mechanism of heme degradation by heme oxygenase.**

**A**

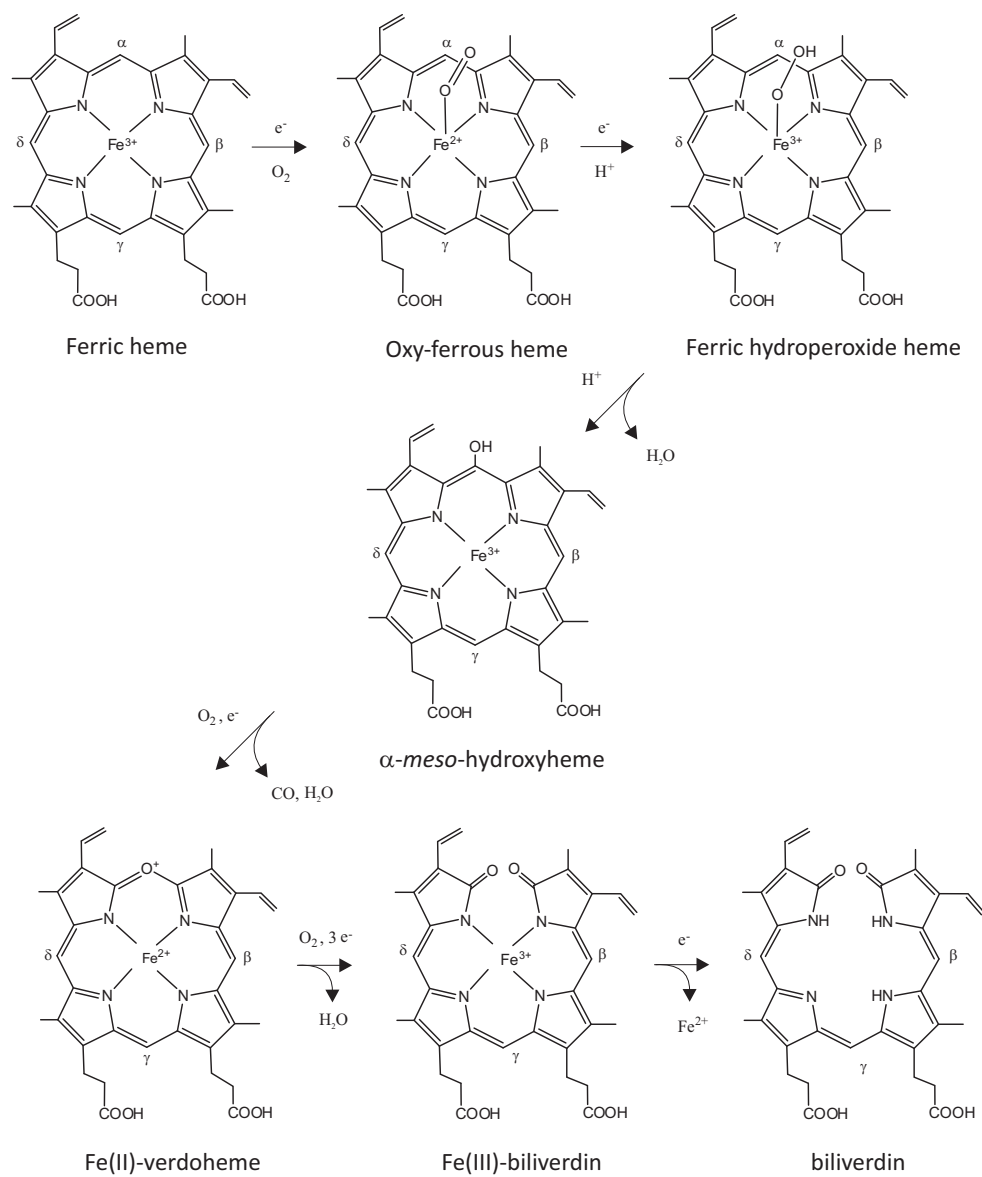

**B**

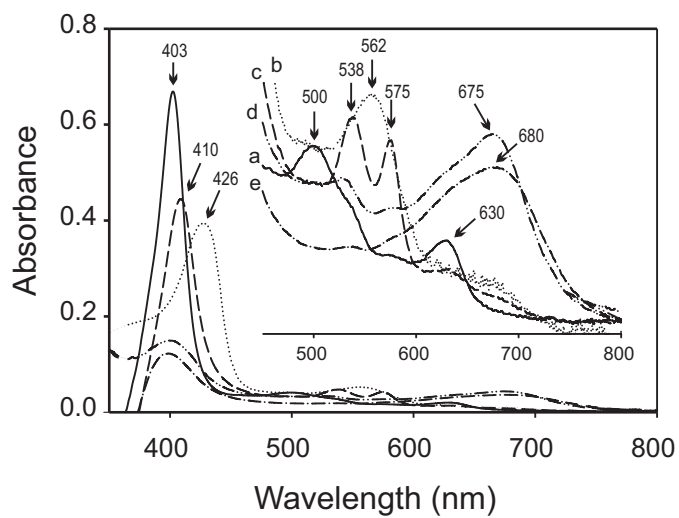

Supplement: S1 Fig — A) Proposed heme degradation pathway in HO. B) Characteristic absorption spectra of complexes formed during heme degradation catalyzed by LepHO. Arrows and numbers indicate wavelengths of characteristic absorption bands of the ferric (—, a), ferrous (···, b), oxyferrous (---, c), verdoheme (- ·· -, d) and biliverdin (- · -, e) complexes. (PDF) [file pone.0182535.s001.pdf]

**S2 Fig. Predicted cartoon model of the LepHO structure**

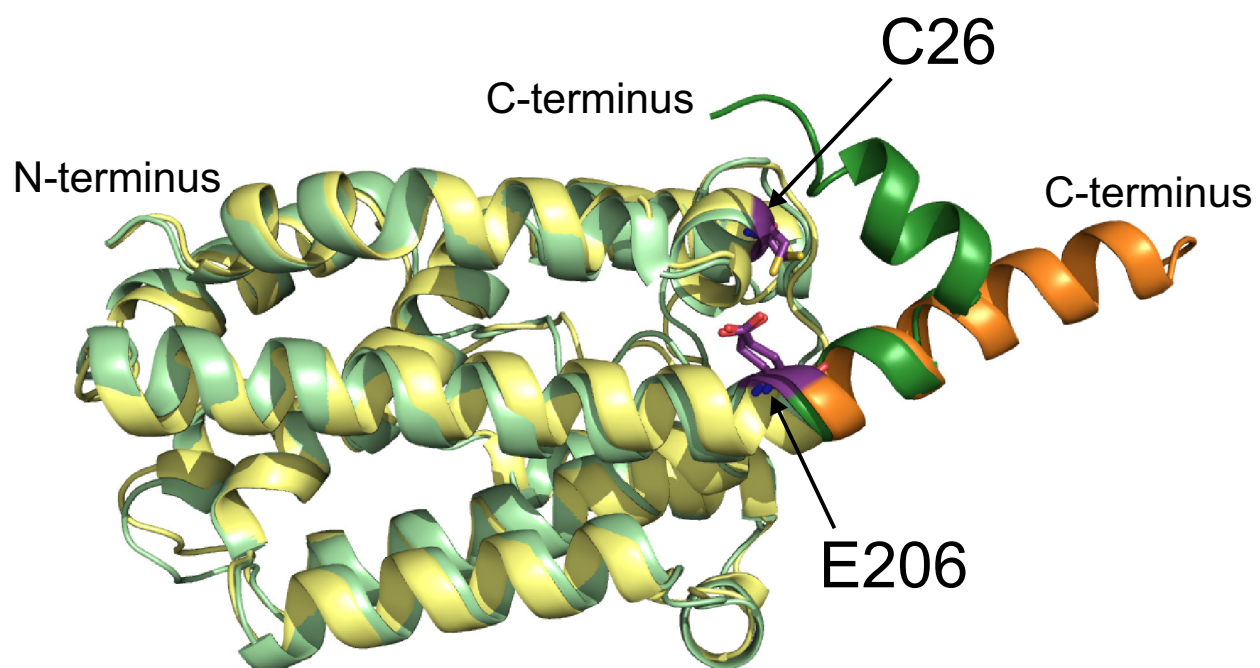

Supplement: S2 Fig — Two models were generated by homology with the I-TASSER program (Roy A, Kucukural A, Zhang Y. I-TASSER: a unified platform for automated protein structure and function prediction. Nat Protoc. 2010;5: 725–38. 10.1038/nprot.2010.5) using the Synechocystis sp. PCC6803 HO-1 (PDB ID: 1WE1, green) and the Synechocystis sp. PCC6803 HO-2 (PDB ID: 1WO1, yellow) as structural templates. A superimposed representation of both models is presented where C26 and E206 residues are depicted in purple. (PDF) [file pone.0182535.s002.pdf]

**S4 Fig. Analysis of purified LepHO variant for crystallization.**

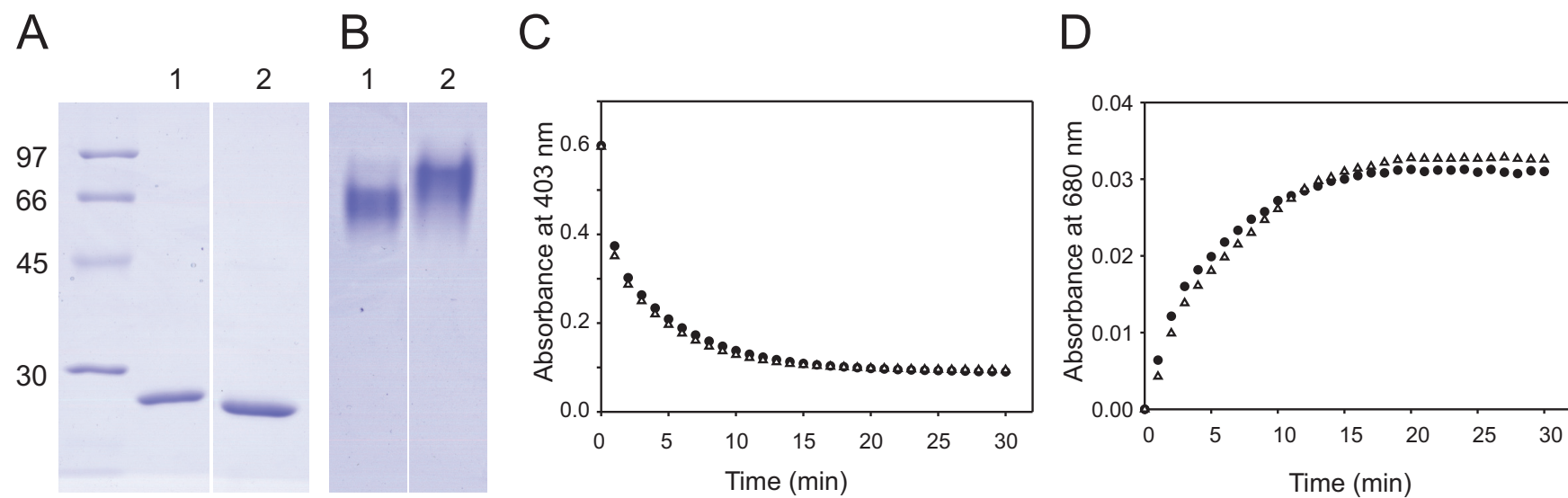

Supplement: S4 Fig — Electrophoresis in 12% polyacrylamide gels in the presence of SDS (A) or under native conditions (B) of the wild-type LepHO (lane 1) or LepHO-C26S-stop (lane 2). Protein standards (kDa) are shown in the first lane in (A). Time dependent absorbance changes at 403 nm (C) and 680 nm (D) were recorded for reactions containing 1 μM LepFNR, 300 μM NADPH, 0.1 mg/ml catalase and 6 μM of wild-type LepHO (●) and LepHO-C26S-stop (Δ) as ferric complexes. The decay of the absorbance at 403 nm indicates heme rupture while increase at 680 nm shows biliverdin formation. (PDF) [file pone.0182535.s004.pdf]

**S5 Fig. Arrangement of the distal G128 and G132 residues in the LepHO structure.**

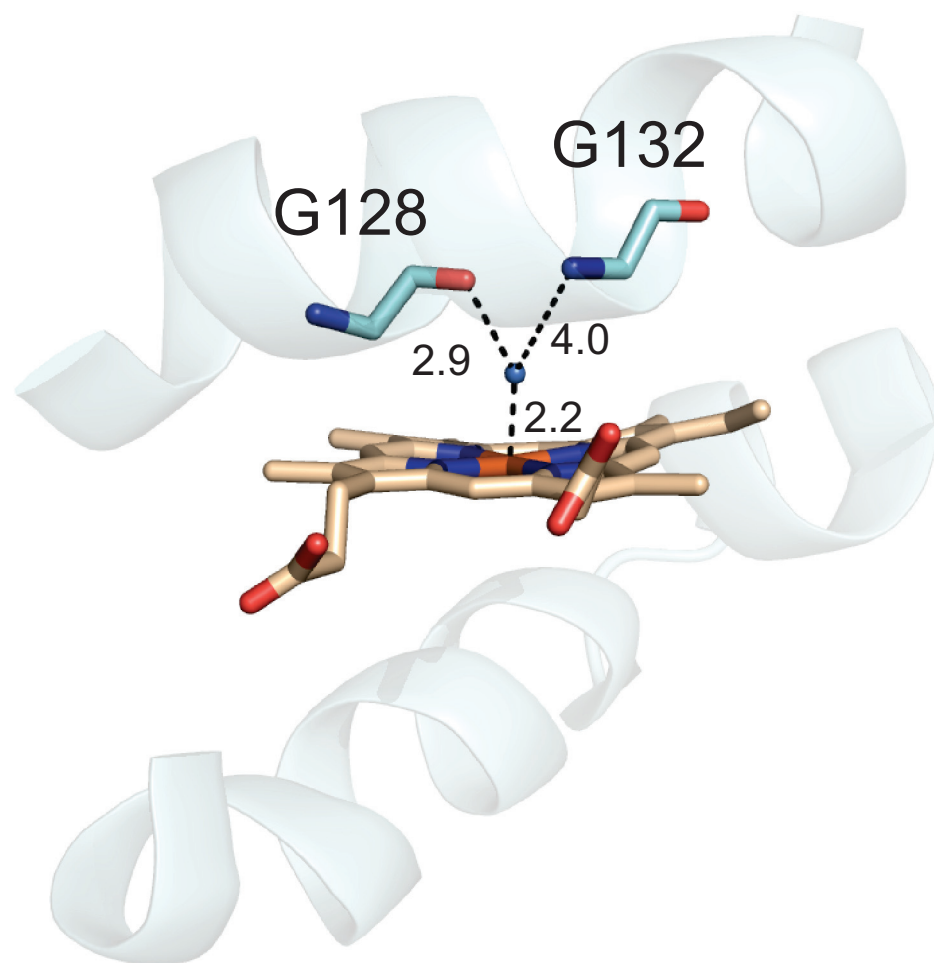

Supplement: S5 Fig — The water molecule located near heme is represented as a blue sphere. Distances are stated in Å and represented as dashed lines. (PDF) [file pone.0182535.s005.pdf]

**S6 Fig. Identification of the reaction product of the heme degradation by the F157I mutant.**

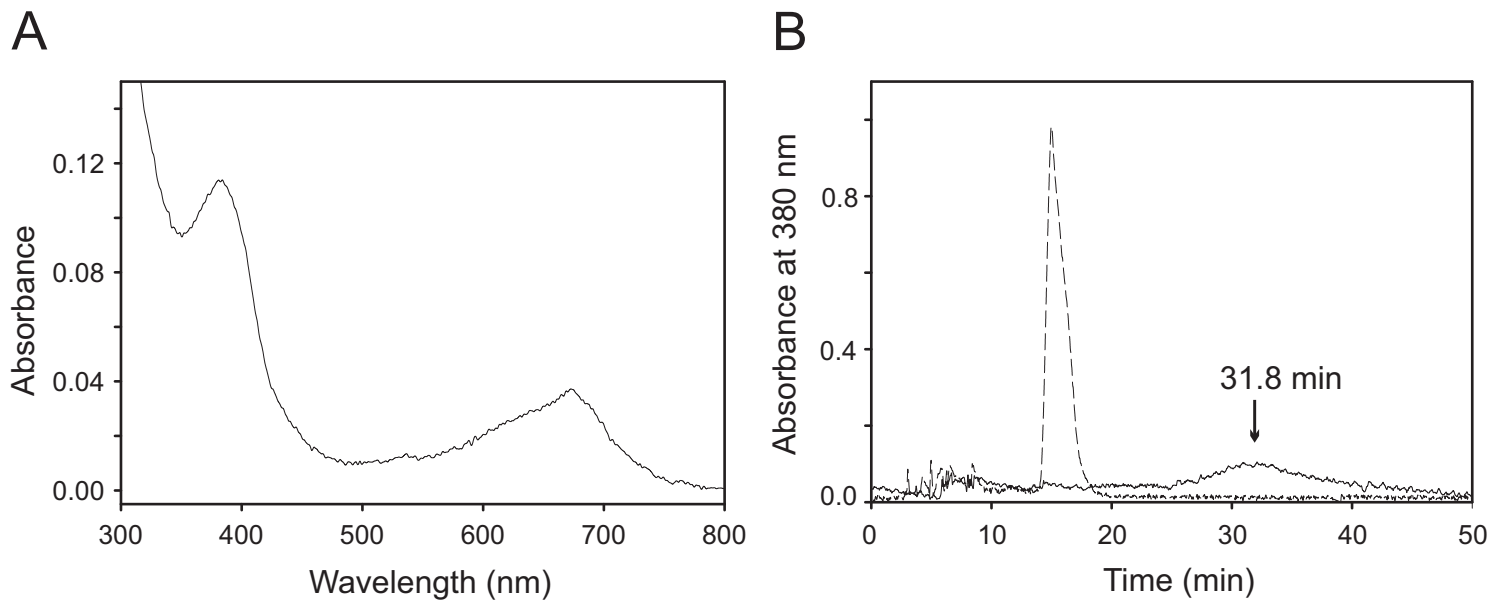

Supplement: S6 Fig — Spectral characterization (A) and HPLC analysis (B). After catalytic conversion of heme by the enzyme, 5% (v/v) pyridine was added, the products extracted with chloroform and analyzed as described in Material and methods. (PDF) [file pone.0182535.s006.pdf]

**S7 Fig. Catalytic turnover of heme by LepHO in the presence of ascorbate.**

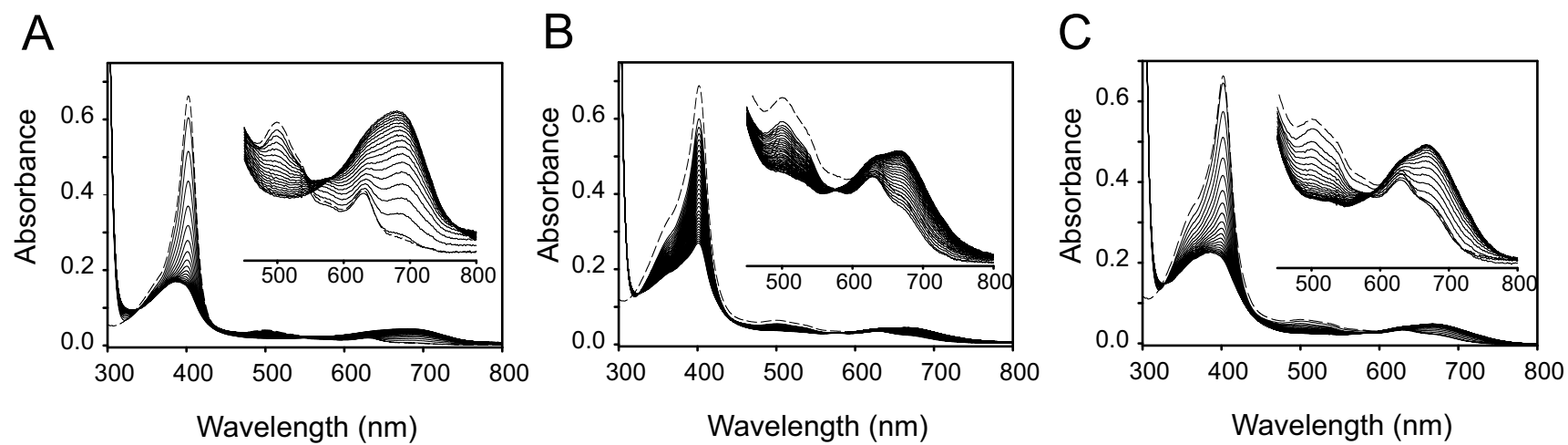

Supplement: S7 Fig — Spectroscopic changes of wild-type LepHO (A), F157I (B) and F157A (C) mutant enzymes before (---) and after (―) addition of 5 mM ascorbic acid. The inset shows an enlargement of the region between 500 and 800 nm. (PDF) [file pone.0182535.s007.pdf]

S8 Fig. Analysis of the LepFNR-LepHO interaction by chemical crosslinking.

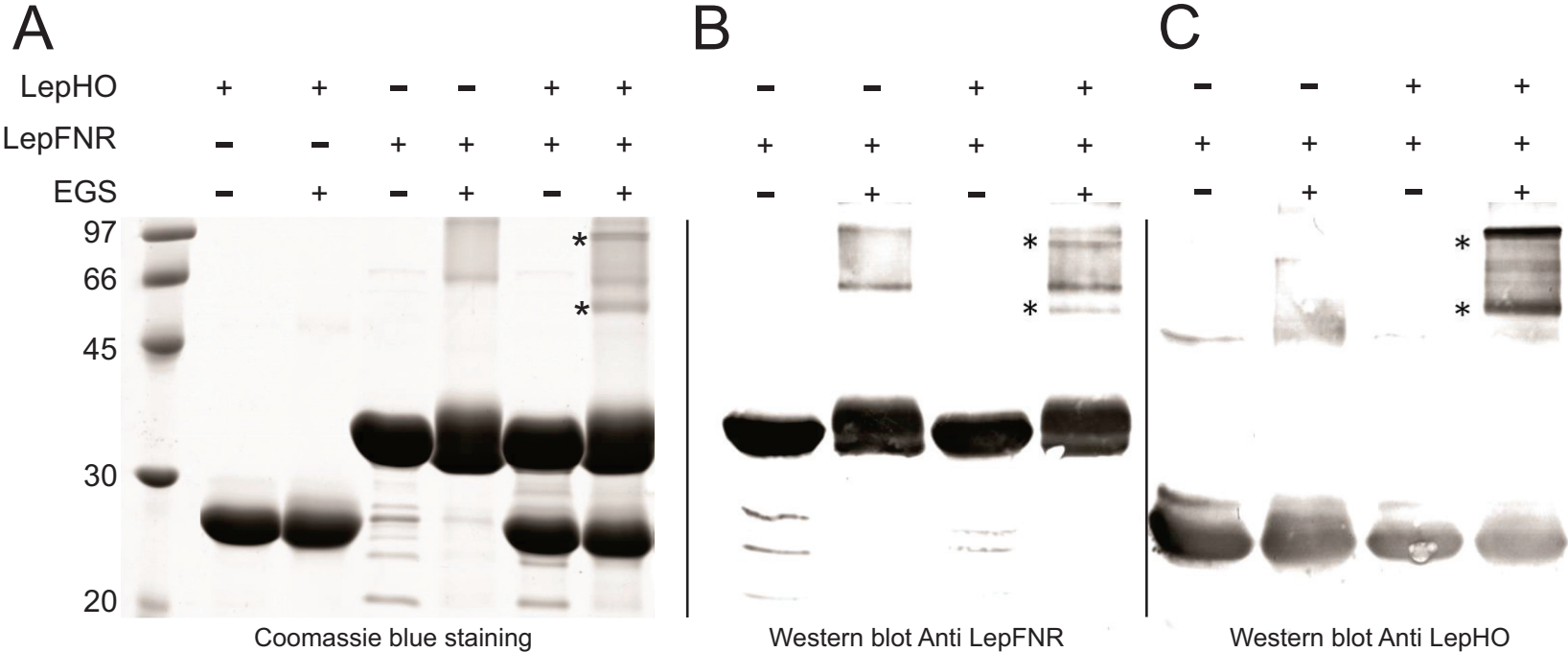

Supplement: S8 Fig — SDS-PAGE (12%) (A) and western blots using Anti LepFNR (B) or Anti LepHO (C) antibodies. Crosslinking reactions were carried out for 30 min using 25 mM EGS in 20 μl of 25 mM HEPES-KOH, pH 7.5, containing LepHO-heme and/or, LepFNR (12.5 μM protein concentration each) as indicated on the figure. Numbers to the left indicate molecular weight markers in kDa. Proteins bands that appear by crosslinking of the complex LepHO-LepFNR (61 and 88 kDa) are indicated with an asterisk. (PDF) [file pone.0182535.s008.pdf]
