# Supplementary material for: Structural and mutational analyses of the Leptospira interrogans virulence-related heme oxygenase provide insights into its catalytic mechanism
Source: PLoS One. 2017 Aug 3;12(8):e0182535. doi: 10.1371/journal.pone.0182535 (PMC5542595; doi:10.1371/journal.pone.0182535)
Supplement: S3 Fig — The relevant residues involved in heme binding (bold, red), mutated or removed for improving crystallizability (bold, green), proposed participating residues in the electron transfer path (bold, purple) and F157 studied in this work (bold, blue) have been highlighted. (PDF) [file pone.0182535.s003.pdf]

S3 Fig. Multiple sequence alignment of LepHO with close homologs.

```
                           8      15  19     26            40
LepHO     ----------MSLATILREGTSEEHKAAESSAFIRCFMKGILEKGTYARHLEAFYYVYES
SynHO1    --------MSVNLASQLREGTKKSHSMAENVGFVKCFLKGVVEKNSYRKLVGNLYFVYSA
SynHO2    ---------MTNLAQKLRYGTQQSHTLAENTAYMKCFLKGIVEREPFRQLLANLYYLYSA
H-HO1     MERPQPDSMPQDLSEALKEATKEVHTQAENAEFMRNFQKGQVTRDGFKLVMASLYHIYVA
                     .*:  *: .*.: *. **.  ::: * ** : :  :   :  :*.:* :

LepHO     MEEELERNKNNLVLKSIYFP-ELYRKNALLEDLQFFYGTWKPNDHQPSVATQDYVQRIRK
SynHO1    MEEEMAKFKDHPILSHIYFP-ELNRKQSLEQDLQFYYGSNWRQEVKISAAGQAYVDRVRQ
SynHO2    LEAALRQHRDNEIISAIYFP-ELNRTDKLAEDLTYYYGPNWQQIIQPTPCAKIYVDRLKT
H-HO1     LEEEIERNKESPVFAPVYFPEELHRKAALEQDLAFWYGPRWQEVIPYTPAMQRYVKRLHE
          :*  : : ::  ::  :*** ** *.  * :** ::**.   :    : . : **.*::

                      123  128 132                       157
LepHO     ISETQPELLAAHSYVRYLGDLSGGQILKKVAARALNLP-EGKGISFYEFPMIQ---DING
SynHO1    VAATAPELLVAHSYTRYLGDLSGGQILKKIAQNAMNLH-DG-GTAFYEFADID---DEKA
SynHO2    IAASEPELLIAHCYTRYLGDLSGGQSLKNIIRSALQLP-EGEGTAMYEFDSLPTPGDRRQ
H-HO1     VGRTEPELLVAHAYTRYLGDLSGGQVLKKIAQKALDLPSSGEGLAFFTFPNIA---SATK
          :. : **** **.*.********** **::   *::*  .* * ::: *  :    .

          167 171                                206
LepHO     FKQNYRTALDSLPVNDSEKQSILAESKQVFLLNQGIFSELEQDLVSAIGKETYDSVLGKG
SynHO1    FKNTYRQAMNDLPIDQATAERIVDEANDAFAMNMKMFNELEGNLIKAIGIMVFNSLTRRR
SynHO2    FKEIYRDVLNSLPLDEATINRIVEEANYAFSLNREVMHDLEDLIKAAIGEHTFDLLTRQD
H-HO1     FKQLYRSRMNSLEMTPAVRQRVIEEAKTAFLLNIQLFEELQELLTHDTKDQSPSRA----
          **: **  ::.* :  :  : :: *:: .* :*  :: :*:  :         .

LepHO     ---------------------
SynHO1    SQGSTEVGLATSEG-------
SynHO2    RPGSTEARSTAGHPITLMVGE
H-HO1     ---------------------
```
