# Supplementary material for: Structural and mutational analyses of the Leptospira interrogans virulence-related heme oxygenase provide insights into its catalytic mechanism
Source: PLoS One. 2017 Aug 3;12(8):e0182535. doi: 10.1371/journal.pone.0182535 (PMC5542595; doi:10.1371/journal.pone.0182535)

**S9 Fig. Evaluation of the spectroscopic characteristics of LepFNR and LepFNRΔK84-G90, and their capabilities to supporting heme degradation by LepHO.**

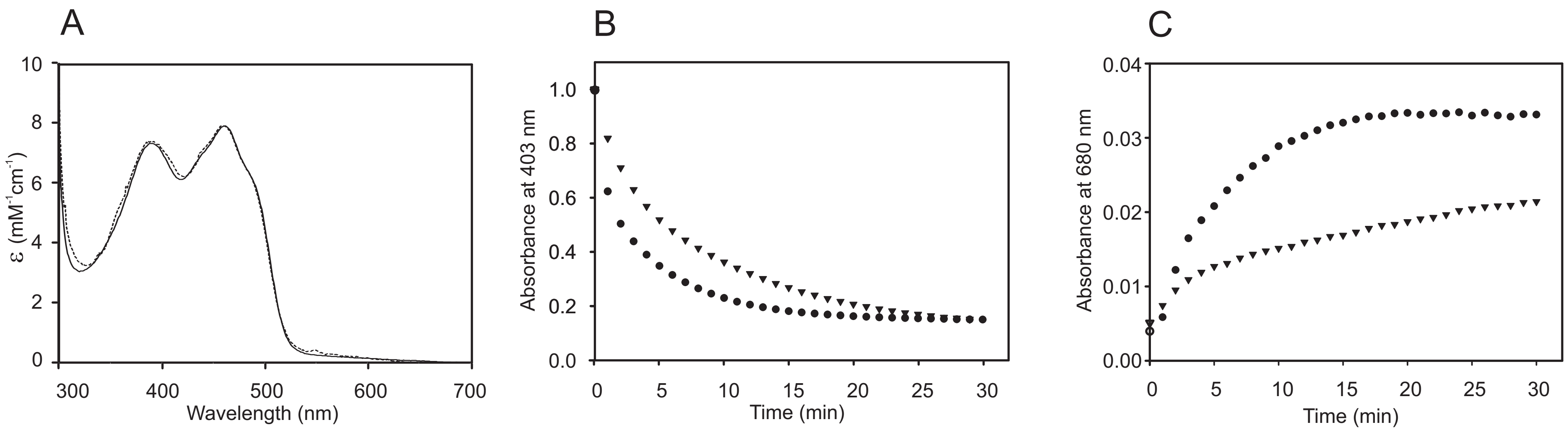

Supplement: S9 Fig — A) Absorption spectra of LepFNR (―) and LepFNRΔK84-G90 (···). Time dependent absorbance changes at 403 nm (B) and 680 nm (C) were recorded for reactions containing 6 μM LepHO, 300 μM NADPH, 0.1 mg/ml catalase and 1 μM of wild-type LepFNR (●) or LepFNRΔK84-G90 (▼). The decay of the absorbance at 403 nm indicates heme rupture while increase at 680 nm shows biliverdin formation. (PDF) [file pone.0182535.s009.pdf]
