# Supplementary material for: Structural and mutational analyses of the Leptospira interrogans virulence-related heme oxygenase provide insights into its catalytic mechanism
Source: PLoS One. 2017 Aug 3;12(8):e0182535. doi: 10.1371/journal.pone.0182535 (PMC5542595; doi:10.1371/journal.pone.0182535)
Supplement: S1 Table — (PDF) [file pone.0182535.s010.pdf]

S1 Table. Nucleotide sequences of synthetic oligonucleotides used for the construction of the *L. interrogans* heme oxygenase and ferredoxin-NADP$^+$ reductase mutants.

LepHO mutant	Oligonucleotide sequences
LepHO-C26S	5' GAAAGTTCTGCTTTCATTCGTAGTTTTATGAAAGGAATATTGG 3'<br>5' CCAATATTCCTTTCATAAAACTACGAATGAAAGCAGAACTTTC 3'
LepHO-stop	5' GGGAATTTTTTCCGAACTGTAACAGGACTTAGTTTCCG 3'<br>5' CGGAAACTAAGTCCTGTTACAGTTCGGAAAAAATTCCC 3'
LepHO-F157A	5' GAATTTCTTTTTACGAAGCTCCTATGATTCAAGAC 3'<br>5' GTCTTGAATCATAGGAGCTTCGTAAAAAGAAATTC 3'
LepHO-F157I	5' GGAATTTCTTTTTACGAAATTCCTATGATTCAAGAC 3'<br>5' GTCTTGAATCATAGGAATTTCGTAAAAAGAAATTCC 3'
LepFNRΔK84-G90	5' CCCGAAAAAAAAGCATATACCGTAAGACTTTATTCC 3'<br>5' GGAATAAAGTCTTACGGTATATGCTTTTTTTTCGGG 3'

The underlined bases indicate the mismatches.
