# Supplementary material for: Structural and mutational analyses of the Leptospira interrogans virulence-related heme oxygenase provide insights into its catalytic mechanism
Source: PLoS One. 2017 Aug 3;12(8):e0182535. doi: 10.1371/journal.pone.0182535 (PMC5542595; doi:10.1371/journal.pone.0182535)
Supplement: S2 Table — (PDF) [file pone.0182535.s011.pdf]

S2 Table. X-ray diffraction data collection and refinement statistics from the LepHO-C26S-stop ferric heme complex

Data collection		
Number of frames		720
Oscillation step (deg)		0.5
Detector distance (mm)		38
Wavelength (Å)		1.5418
Exposure per frame (s)		120
Indexing and scaling		
Cell parameters	a (Å)	37.64
	b (Å)	58.79
	c (Å)	86.70
	$\alpha = \beta = \gamma$ (deg)	90.00
Space group		$P2_12_12_1$
Resolution limit (Å)		1.73
Number of unique reflections		20079
Average multiplicity [a]		13.2 (10.0)
$\langle I/\sigma(I) \rangle$		20.8 (2.6)
R_{meas}		0.129 (1.178)
R_{pim}		0.037 (0.583)
$CC_{1/2}$		0.998 (0.613)
Completeness (%)		96.7 (89.3)
No. of chains per asymmetric unit		1
Solvent content (%)		38
Overall B-factor (Wilson plot, Å^2)		17
Refinement		
Resolution range (Å)		31.70-1.73
Number of protein atoms		1687
Number of ligand atoms		43
Number of water molecules		219
R		0.186
R_{free}		0.222
Rms deviations from ideal values [1]		
Bond lengths (Å)		0.010
Bond angles (deg)		1.0
Average B-factor (Å^2)		16
MolProbity validation [2]		
Clashscore		5.40
MolProbity score		1.45
Ramachandran plot		
Favored (%)		98.5
Allowed (%)		1.5
Disallowed (%)		-
Protein Data Bank deposition		
PDB code		5KZL

[a] Values in parentheses correspond to the highest resolution shell (1.73-1.83 Å)

1. Engh RA, Huber R. Accurate bond and angle parameters for X-ray protein structure refinement. Acta Crystallogr Sect A. 1991;47: 392–400. doi:10.1107/S0108767391001071
2. Chen VB, Arendall WB, Headd JJ, Keedy DA, Immormino RM, Kapral GJ, et al. MolProbity: all-atom structure validation for macromolecular crystallography. Acta Crystallogr D Biol Crystallogr. 2010;66: 12–21. doi:10.1107/S0907444909042073
