# Supplementary material for: Structural and mutational analyses of the Leptospira interrogans virulence-related heme oxygenase provide insights into its catalytic mechanism
Source: PLoS One. 2017 Aug 3;12(8):e0182535. doi: 10.1371/journal.pone.0182535 (PMC5542595; doi:10.1371/journal.pone.0182535)
Supplement: S3 Table — (PDF) [file pone.0182535.s012.pdf]

S3 Table. Volume and depth of heme oxygenase prosthetic group pockets from different organisms

Protein (PDB code)	Organism	Volume ($Å^3$)	Average pocket depth ($Å^3$)
LepHO (this work)	*L. interrogans*	418	3.58
HO-1 (1WE1)	*Synechocystis* sp.	417	5.07
HO (1IW0)	*C. diphtheriae*	406	4.73
HO-1 (1N45)*	*Homo sapiens*	348	5.55

* the calculated volume corresponding to the chain of the structure showing a closed conformation. Volume and average pocket depth were calculated using POCASA (V 1.1) software.[1]

1. Yu J, Zhou Y, Tanaka I, Yao M. Roll: A new algorithm for the detection of protein pockets and cavities with a rolling probe sphere. Bioinformatics. 2009;26: 46–52. doi:10.1093/bioinformatics/btp599
